# Fleeting Perceptual Experience and the Possibility of Recalling Without Seeing

**DOI:** 10.1038/s41598-020-64843-2

**Published:** 2020-05-22

**Authors:** William Jones, Hannah Pincham, Ellis Luise Gootjes-Dreesbach, Howard Bowman

**Affiliations:** 1 Centre for Cognitive Neuroscience and Cognitive Systems, University of Kent, Canterbury, UK; 2 South Eastern Sydney Local Health District, Sydney, New South Wales Australia; 3 Department of Psychology, University of Birmingham, Birmingham, UK; 4 Point Estimate Limited, Ellesmere Port, Cheshire, UK

**Keywords:** Attention, Consciousness, Perception, Network models

## Abstract

We explore an intensely debated problem in neuroscience, psychology and philosophy: the degree to which the “phenomenological consciousness” of the experience of a stimulus is separable from the “access consciousness” of its reportability. Specifically, it has been proposed that these two measures are dissociated from one another in one, or both directions. However, even if it was agreed that reportability and experience were doubly dissociated, the limits of dissociation logic mean we would not be able to conclusively separate the cognitive processes underlying the two. We take advantage of computational modelling and recent advances in state-trace analysis to assess this dissociation in an attentional/experiential blink paradigm. These advances in state-trace analysis make use of Bayesian statistics to quantify the evidence for and against a dissociation. Further evidence is obtained by linking our finding to a prominent model of the attentional blink – the Simultaneous Type/Serial Token model. Our results show evidence for a dissociation between experience and reportability, whereby participants appear able to encode stimuli into working memory with little, if any, conscious experience of them. This raises the possibility of a phenomenon that might be called sight-blind recall, which we discuss in the context of the current experience/reportability debate.

## Introduction

The ability to seperate functionally independent mental processes, and to be able to describe this seperation – or lack thereof – is critical to modern cognitive neuroscience. Of these problems of independence, the distinction between the subjective experience of the character of a stimulus (the “phenomenological awareness” of it) and the ability to objectively report on it (the “access consciousness” of it) has been one that has been particularly hotly contested. Block^1^ is a notable proponent of a distinction between the two, arguing that it is possible to experience stimuli without being able to access them, and thus report on that experience. The believed locus of phenomenological awareness is iconic memory, initially, on the basis of the Sperling paradigm^2^, with others supporting the concept of phenomenological awareness to varying degrees on the basis of experiments on Kanizsa triangles^3^, other, modified versions of the Sperling paradigm^4^, and short term memory experiments^5^. However, despite this large body of supporting literature, the theory is contested; for example, Dehaene and co-workers^6^ have challenged this theory on the basis of change blindness, while others have pointed out that certain changes to the Sperling paradigm seem to compromise some key results^7^.

**Figure 1:**
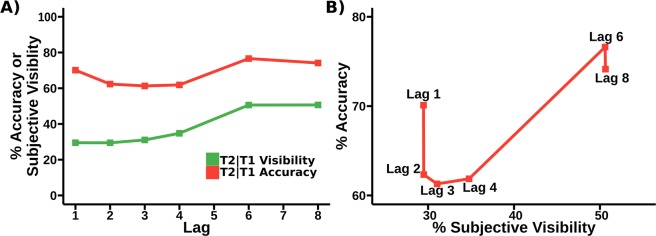
(**A**) Results from^11^, comparing accuracy and subjective visibility across lags in the attentional blink. The T2 visibility curve demonstrates what Pincham and Bowman term the Experiential blink of subjective report. (**B**) State-trace plot comparing T2|T1 accuracy and T2 visibility from (**A**). Note the apparent non-monotonicity of the relationship between accuracy and visibility. (Note, the T2|T1 blink curve here shows some very minor differences to that presented in^11^. This is because T2 accuracy in the original paper was mislabeled and in fact presented the accuracy of the conjunction of T2 and T1, whereas here we display the conditional probability of T2 given T1. None of the findings in^11^ are impacted by this difference).

A paradigm that is well placed to shed light on this topic, and has been used previously^8^ to explore the all-or-none nature of subjective experience, is the attentional blink. The attentional blink is a phenomenon seen during RSVP (Rapid Serial Visual Presentation) in which participants frequently fail to detect a second target for a short time after the presentation of an encoded first target; see T2|T1 accuracy in Fig. 1^9,10^. Recently, Pincham *et al*.^11^ noted that the temporal pattern of T2 visibility (which they called the experiential blink) is dissimilar to that of report accuracy (i.e. the classical attentional blink) and raised the possibility that this finding represents two distinct processes. However, having the tools to elicit dissimilar patterns of behaviour is not the same as being able to determine whether the cognitive processes that underlie them are distinct. Tackling such problems is usually performed by looking for functional dissociations. These arise when we find variables that allow us to independently modify performance on two separate tasks, providing putative evidence that the cognitive processes embodied by the tasks are in some way separate. Such dissociation logic has been widely applied, and made an important contribution to the investigation of functional independence in the mind in such diverse sub-fields as short and long term memory^12^, word comprehension^13^ and consciousness^14^.

In the context of our question, there are many who have claimed that the experience or awareness of a stimulus and its reportability are doubly dissociated. As previously discussed, in the direction of awareness without report, we have the “phenomenological consciousness” of Block. In the opposite direction, there exist several paradigms that seem to provide evidence for modulation of behaviour without awareness, for example continuous flash suppression^15^, visual masking^16^, blindsight^17^, or episodic face recognition^18^. However, we would argue that these paradigms provide evidence for a weaker claim than reportability without awareness; that of *influence* without experience. In every case, the identity of the unexperienced stimulus is not directly reportable, it merely influences the report of, or response to, something else. In contrast, the criterion for a true demonstration of reportability without awareness would be of free recall of a stimulus identity in the absence of awareness, which, if definitively demonstrated, would be both striking and surprising.

Regardless, even if a double dissociation of the required kind between experience and reportability was widely agreed to exist, there has been a long standing debate about the use of double dissociations as a measure by which to assess functional differentiation^19–21^. In this work, we adopt an alternative method to traditional dissociation logic. This alternative suggests that a dissociation arises, given certain assumptions, when it is not possible to demonstrate a monotonic relationship between task performances. In the context of the attentional blink, there is evidence that such non-monotonicity exists between accuracy and subjective visibility report^11^ (see Fig. 1), and one of the main contributions of this paper is to provide quantitative evidence for such an effect.

In order to provide statistical quantification, a method called state-trace analysis is typically employed. State trace analysis examines the monotonicity of data, across a state-trace plot in which our two task performances form the axes. In this work, we follow Prince, Brown and Heathcote^22^ and Davis-Stober *et al*.^21^ in advocating the use of a Bayesian approach to the analysis of these problems. The main reason for this is that we are solving a model comparison problem: comparing whether a non-monotonic or monotonic model best fits our data. Strictly speaking, a classical statistics approach would not enable us to find evidence for a non-monotonic outcome, since it would naturally take the role of the null. For a more detailed discussion on the various potential choices of statistical methods and their respective virtues, see^22^.

While dissociations can tell us about specific effects, placing findings in larger theoretical context is pivotal to the forward progress of science, especially when the theory is encapsulated in a computational model. In particular, a theoretical interpretation of the data from^11^ may be that items are encoded into working memory simultaneously, but only experienced serially. In combination with state-trace analysis, this allows us to explore not only the direction of the effect, but also some plausible mechanisms by which it may arise. In terms of specific models, the Simultaneous Type/Serial Token^10^ model is well placed to explore this question: it models data in the relevant context (the attentional blink), and naturally deals with the difference between simultaneity and seriality.

In this paper, we make two original contributions. We first apply Bayesian state-trace analysis to the results of our attentional blink experiment in which we collected both report accuracy and subjective visibility (see Fig. 1), and compare the respective evidence for a monotonic and a non-monotonic relationship between the two measures. Secondly, we explore our results in the context of the Simultaneous Type/Serial Token (STST) model. Since the STST model does not natively deal with subjective experience, one of the contributions of this paper is development of a simple method by which this might be incorporated into the model. Given this method, we then compare the behavioural and EEG data that the model predicts to the human data from^11^, and the results from our state-trace analysis.

## The Attentional Blink Paradigm

**Figure 2:**
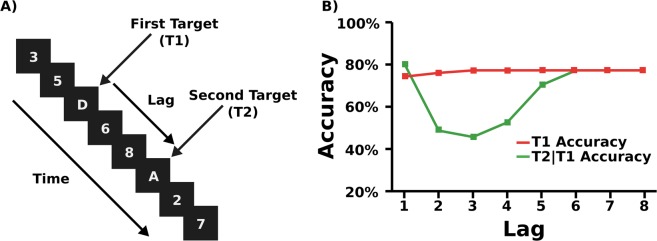
(**A**) A typical attentional blink RSVP stream. Participants are instructed to report the two letters at the end of the stream. (**B**) Example illustration of expected accuracy for T1 and T2|T1 at each lag during a typical attentional blink study with a Stimulus Onset Asynchrony (SOA), the amount of time between the onset of each stimulus, of 80–120 ms.

Rapid serial visual presentation (RSVP) is a technique in which multiple stimuli are presented rapidly, one after the other in a fixed location. Typically, this stream of stimuli is composed of one or more targets to be detected or identified and a number of distractor stimuli to be ignored. The attentional blink (AB) is a deficit in performance on a second target when more than one target is to be identified^9,10^. It arises approximately 100–500 ms after the presentation of the first target, when it is successfully encoded. Typically, the AB is elicited using alphanumeric stimuli, but images, letters, digits or words will all elicit the blink. For an example of a typical attentional blink RSVP stream, see Fig. 2.

The main parameter of the attentional blink is the relative serial positions at which the two targets are presented, known as lag, for example, at Lag 1 there are no intervening distractors between the targets, while at Lag 2, the two targets are separated by one intervening stimulus. The main attentional blink result is typically plotted as T2|T1 accuracy (second target accuracy, given the first target was correct) against lag. Excluding Lag 1, typically, when the two targets are close, accuracy is significantly reduced compared to recovery baseline (lags 7 and 8). A typical blink is shown in Fig. 2(B). Performance at Lag 1 is above the deepest point in the blink. This is known as Lag 1 sparing, and is itself a robust result of the attentional blink^23^.

There has been extensive exploration of the attentional blink with respect to accuracy of report, but much less exploration of subjective visibility report in the attentional blink^8,11,24,25^. As we have discussed, the attentional limitations of the blink make it ideal for exploring dissociation between accuracy in reporting a stimulus and the strength of its conscious experience. Indeed^11^, mapped subjective report to lag, finding a blink of subjective experience, the so called Experiential Blink, akin to that of reportability, but without Lag 1 sparing. The results of this experiment are shown in Fig. 1.

## Functional dissociations and reversed associations

As mentioned previously, the functional dissociation is a technique that has been widely implemented across the fields of psychology and neuroscience as a marker of the functional distinctness of mental processes. There are several types of functional dissociations, but all arise when one is able to independently modify performance on a set of one or more tasks without affecting performance on other tasks in the set. The ability to differentially affect behaviours on different tasks is seen as evidence that the mental processes underlying them are in some way functionally separate. However, despite their wide use in the literature, it has been argued that while dissociations are certainly indicative, they do not strictly provide either a necessary or sufficient basis for determining the separation of mental processes^19–21^. Broadly, it has been proposed that it is possible to construct cases in which dissociations exist but separate mental processes do not^19–21^, and to create cases in which there are separate mental processes without dissociations. For an overview of these arguments, and a demonstration of how such behaviours can be constructed, see^26^.

Regardless which side of this debate one stands, an alternative measure exists for which it is certain these issues will not arise: the reversed association proposed by^20^. The reversed association models the cognitive function that dissociations are trying to evaluate as a latent variable determining the relationship between a given task and task performance. It then assumes that, while the relationship between this latent cognitive function and task performance may not be proportional, it may at least be assumed to be monotonic in some direction^27^. Given this assumption of monotonicity between cognitive function and task performance, any tasks that share a single underlying cognitive process must then, by necessity, also share a monotonic relationship between their respective task performances. Therefore, under these assumptions, a non-monotonic relationship between task performances is sufficient to demonstrate a dissociation, this is our reversed association. Note that the opposite does not apply, a monotonic relationship is not sufficient to demonstrate that the cognitive functions underlying the two lack a dissociation. In order to undertake statistical inference for a reversed association, we turn to Bayesian statistics.

## Quantifying the results – the Bayesian method

**Figure 3:**
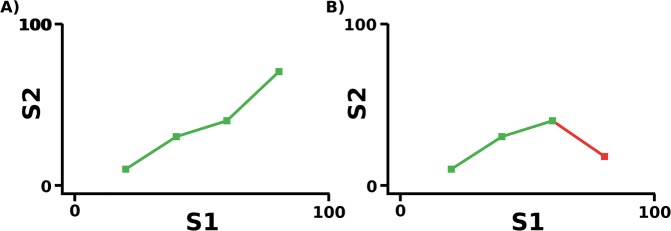
(**A**) Example of a monotonic state-trace plot across 4 levels of a dimension factor D. It is possible to draw a monotonic (increasing) curve joining all points, therefore the relationship between the levels of the state factor is monotonic. (**B**) Example of a non-monotonic state-trace plot across 4 levels of a dimension factor D. The point furthest to the right makes drawing either a monotonically increasing or monotonically decreasing curve impossible, therefore the relationship between the levels of the state factor is non-monotonic.

We describe state-trace analysis informally in terms of a state-trace plot, e.g. Fig. 3. We have a state factor consisting of our two tasks, with the performance on each task forming an axis on our graph. We then plot on this graph each level of our dimension factor, the variable that we are varying across our tasks. If we can draw a monotonically increasing (or decreasing) curve joining all the levels of our dimension factor, the relationship between our task performances across our variable is monotonic. In all other cases, it is non-monotonic. In the context of our attentional blink experiment, identity report and judging visibility are our two tasks so they give us our state factor, and the lags are the measure that we are varying across both tasks, so they give us our dimension factor. Plotting report accuracy on one axis and visibility on the other, we are trying to determine whether it is possible to draw a monotonic curve joining the data across each of our lags.

More formally, we have some state factor with two levels *S* = {*S*_1_, *S*_2_}, forming the state space over which we examine our question of interest, and some dimension space *D* = {*D*_1_, …, *D*_*n*_}, a manipulation we are performing across it. When concerned with monotonicity versus non-monotonicity, we wish to see if the ordering of the levels of our dimension factor are either the same or the reverse of one another across each of the two axes of our state factor. If this is possible, we diagnose monotonicity, and if it is not possible we do not. Often, we also introduce a trace factor, but in our case, a trace factor is not required and we therefore exclude it from further discussion. Overall, we must consider each combination of *Q* = *D*! orderings for each axis and *Q*^2^ joint orderings. A visual example of both monotonic and non-monotonic state-trace plots can be found in Fig. 3.

At this point, the set of *Q*^2^ joint orderings corresponds to the whole space of possible configurations of the state-trace graph, and currently it can be divided into two different partitions. These are the non-monotonic orderings and the monotonic orderings. With respect to our Bayesian statistics, we are attempting to choose between the monotonic model consisting of all monotonic orderings, and our non-monotonic model consisting of all other (non-monotonic) orderings. To do this, we calculate a Bayes factor expressing how much the data has changed our preference between our two models. This is the measure of the ratio of evidence for each model. Explicitly, denoting our data as y, the prior probabilities *P*(*x*) where *x* = *M* or *NM* as *π*_*M*_ and *π*_*NM*_ for the monotonic and non-monotonic models respectively, and the posterior probabilities *P*(*x*|*y*) where *x* = *M* or *NM* as ${\pi }_{M}^{(y)}$ and ${\pi }_{NM}^{(y)}$, we calculate the Bayes factor as:

$$B{F}_{M/NM}=\frac{{\pi }_{M}^{(y)}}{{\pi }_{NM}^{(y)}}/\frac{{\pi }_{M}}{{\pi }_{NM}}$$

We calculate our posterior using the library provided in^21^. We follow^21^ in referring to this calculation as *BF*_*N*/*NM*_, the bayes factor comparing the monotonic versus non-monotonic models.

Currently, we make use of a completely uniform prior, effectively assuming all possible orderings of the lags across the levels of the state factor are equally likely. In many data sets, including our own, this is clearly not true – we, for example, have strong prior expectations about the behaviour of the attentional blink. Previous work has approached this problem by using the prior to assert that certain constraints on the behaviour in the data are true. For example, in^21^ the authors pre-suppose that dual task performance will always be worse than single task performance in their analysis of a data set from^28^. However, while we have expectations about the behaviour in the attentional blink, setting specific ordinal qualifications of behaviour across lags in a similar manner is non-trivial. While we wish to take advantage of as much prior knowledge as possible, the behaviour of the attentional blink is variable, and it is well established that setting a poor prior can compromise the integrity of results^29^. As well as setting a prior based on previous literature, we also therefore make use of an empirical prior method to derive a suitable prior. This method takes the set of constraints on the prior identified from the literature, and reduces the set to one that accurately fits the data, using a measure of the validity of constraints orthogonal to the contrast of interest. Details of this method can be found in Supplementary Material. We denote the validity of a prior calculated using this method as *BF*_*D*/*N*(*D*)_, and similarly any Bayes factor calculated from a prior that accounts for information on our dimension axis (whether generated from our empirical priors method or not) as *BF*_(*M*/*NM*)|*D*_.

We must also consider how to apply this type of analysis across a group of participants. Notably, state-trace analysis does not work well with approaches based on averaging. In particular, it is possible both to average multiple non-monotonic datasets into a monotonic dataset, and multiple monotonic datasets into a non-monotonic one. A simple alternative analysis is the grouped Bayes factor introduced by^22^. This method treats each of our participants (of which there are M) as independent from one another and calculates the group Bayes factor as the product of each individual Bayes factor:

$$GBF={\prod }_{i=1}^{M}\,B{F}_{i}$$

As long as participants are independent samples and the results are reasonably homogeneous (not, for example, being driven by a single outlier), this grouped Bayes factor is a good summary of the group level effect. This will be the case in the data we analyse with one exception that will be discussed seperately.

## STST model

In addition to the methods of state-trace analysis, we explore the potential dissociation of subjective experience and report accuracy through modelling. Specifically, we investigate the hypothesis that the differences in behaviour in the data from^11^ that we analyse in this paper are the result of the systems of subjective experience and working memory encoding being dissociated. We suggest that stimuli are experienced in a serial manner (reflecting the unitary nature of consciousness), but simultaneously encoded into working memory. The Simultaneous Type/Serial Token (STST) model^10^ is in a uniquely strong position to explore this, though the model does not natively deal with subjective experience. In this section, we explore a simple set of additions to the STST model that allow it to read out a measure of subjective experience in addition to reporting accuracy. Before this however, we briefly summarise the workings of the Simultaneous Type/Serial Token model.

**Figure 4:**
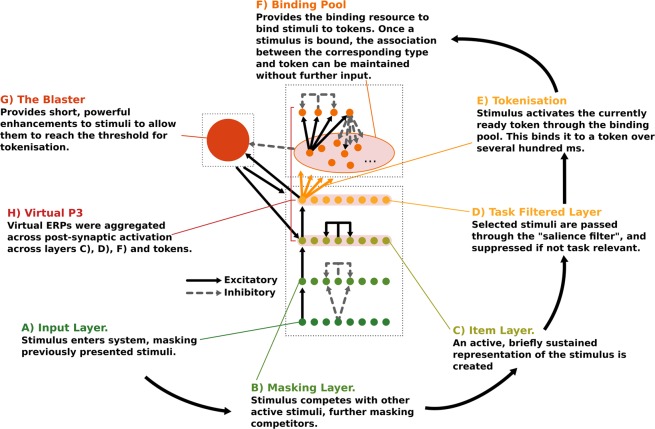
(**A**) Input Layer. Stimuli enter the system through this layer. As well as providing input, this layer implements backward masking through inhibitory connections to all other stimuli in the masking layer. (**B**) Masking Layer. Simulates further masking dynamically through lateral inhibitory connections to all other stimuli. These lateral inhibitory connections are weaker than the forward ones from the input layer, such that backward masking is stronger than forward masking. (**C**) Item Layer. Creates a temporary representation of a stimulus through self-reinforcing connections. (**D**) Task Filtered Layer. Implements a “salience filter” to filter out task irrelevant stimuli, by enhancing task relevant stimuli, and suppressing others. (**E**) Tokenisation. When a stimulus has reached an appropriate level of activation, it excites the currently ready token through the binding pool. In a process that takes several hundred ms, the token is bound to the type. Once this binding has occurred, the type-token connection can be maintained without any further input. (**F**) The Binding Pool. Contains the binding resources that enable stimuli to bind to tokens. (**G**) The Blaster. Provides a short, powerful enhancement to items in the item and task filtered layers when there is sufficient activation in the task filtered layer to indicate the ‘detection' of a target and warrant the onset of tokenisation. While the tokenisation process is ongoing, a powerful inhibitory signal from the binding pool prevents the blaster firing again. (**H**) Virtual P3. A virtual P3 can be generated from the STST model from the excitatory post synaptic potentials of the item layer, the task filtered layer, and a subset of the tokens and binding pool (the token gates and the binder gates).

The STST model, see Fig. 4, is a two stage model that builds on a type/token distinction to simulate how items are bound into temporal contexts. In this definition, the type of a stimulus encompasses all of its instance invariant properties: the features that do not change between occurrences. Take the letter K for example; parts of its type are its semantic features (e.g. it’s a letter, it’s after J in the alphabet) and its visual features (e.g. its shape and colour). Conversely, a token represents a specific episodic occurrence of a type e.g. where it occurred in time relative to other items. In the STST model, types are processed in parallel, with many types simultaneously but fleetingly represented, and it is the act of sequentially binding a type to a token that creates a solidified representation in working memory.

The first stage of the model concerns the types and consists of four layers supporting different aspects of visual processing: the input layer, the masking layer, the item layer and the task-filtered layers. The second stage of the model governs the tokenisation process, and consists of the binding pool and the tokens. Items first arise in the input layer, and then pass through the masking layer, which implements masking, and would most naturally be associated with iconic memory^2^. From here, items enter the item layer, which creates a brief, self-sustained representation. Then, the final layer of the stage: the task filtered layer, provides a salience filter that excites task relevant nodes while inhibiting others. From the task filtered layer, sufficiently active items can activate tokens through the binding pool, and become bound to them through a tokenisation process. This tokenisation process takes several hundred milliseconds, though it is shorter for more active items. In order to reach sufficient activation to achieve this binding however, most stimuli will need to benefit from the blaster. When an item becomes sufficiently active in the task filtered layer, the blaster provides a brief, powerful enhancement to the entire task filtered and item layers that allows items to reach the threshold for tokenisation. During this process, a powerful inhibitory signal holds the blaster low to prevent it from re-firing and corrupting the tokenisation process: it is this inhibition of the blaster that generates the attentional blink. A walk through of how an individual item becomes encoded into working memory can be seen in Fig. 4.

Through these mechanisms, the Simultaneous Type/Serial Token model creates an account of working memory encoding in which types are processed simultaneously, but due to the way the blaster and the tokenisation process work, types can only be bound in serial. There exists a computational model of STST from which it is possible to generate both behavioural data, and also “virtual” ERP’s^30,31^ that closely mimic the results from human participants. It is an ideal choice for modelling the data which we are exploring, because it is specific to the paradigm we are using (the attentional blink), and it already deals naturally with the difference between simultaneity and seriality.

As discussed, the published STST model does not however, deal with subjective experience, and one of the contributions of this paper is to propose and implement a system by which this can be obtained. However, very many, and often any behaviours can be obtained from a model with sufficient modification and parameter adjustments^32^. In order to make the fairest possible assessment of the hypothesis in question, the dissociability of subjective experience and report accuracy during the attentional blink, we therefore limited ourselves in two ways in our modelling. Firstly, we would attempt to build on top of the existing model to provide a new “readout” without changing the existing model in any way. Secondly, this readout must be simple; ideally arising from one or two principles.

**Figure 5:**
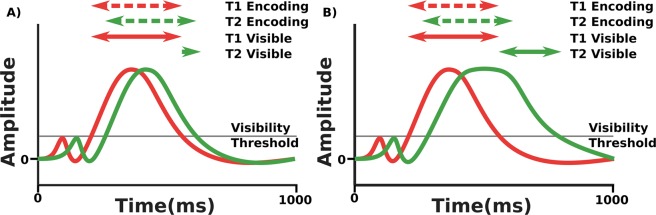
(**A**) Seriality of experience in the SESE model. In (**A**), though the amplitude of the response of both stimuli is the same, the duration of the experience of the second stimulus is greatly reduced because it cannot be experienced until the first stimulus falls below the threshold. Comparatively, in (**B**), the response amplitude of both stimuli is the same, although the T2’s activation trace is longer with a slightly delayed onset, consequently they are both experienced for similar durations.

The result of these conditions is the following model to encapsulate serial experience: Subjective visibility is indexed by the strength of the P3 ERP component. When an item is above a given amplitude (the threshold of subjectivity), it is being “subjectively experienced” and when it is below, it is not. Additionally, this experience is serial. If the individual activation traces for two items are both above the threshold, then the second item cannot be experienced until the first one falls below the threshold. For an illustration of this, see Fig. 5. Specifically, the strength of an item’s subjective experience is the duration for which its activation trace exceeds the threshold of subjectivity, subject to no other stimulus already being above the threshold. In this manner, a system allowing a subjective experience that is exclusively serial in manner is created, with only one addition on top of the existing model. We call this readout-enhanced STST model, the Simultaneous Encoding, Serial Experience model (SESE). In order to evaluate the success of this modified STST model, we will compare its behavioural output to that of human participants and the virtual ERPs it generates to human EEGs in the data from^11^. This specification of subjective experience mandates a change to how we calculate the grand P3 ERPs from the model. The ERPs generated from the model in^31^ are calculated by summing all components together. In this model, when a first target’s activation trace crosses the threshold, it starts contributing to the P3, however, the activation traces of other targets do not contribute to the P3. A more detailed desciption of how virtual ERPs can be obtained from the model is available in Supplementary Material Section D.

## Predictions and Validation

Our current model makes some strong predictions, some of which cannot be immediately validated through the analysis of our first, dataset which we distinguish by referring to it as the colour-marked task (since in the task, the T1 is colour marked, which is not the case in the letters-in-digits task that we introduce shortly). In this section, we discuss these analyses and propose several further analyses to support our hypothesis.

**Figure 6:**
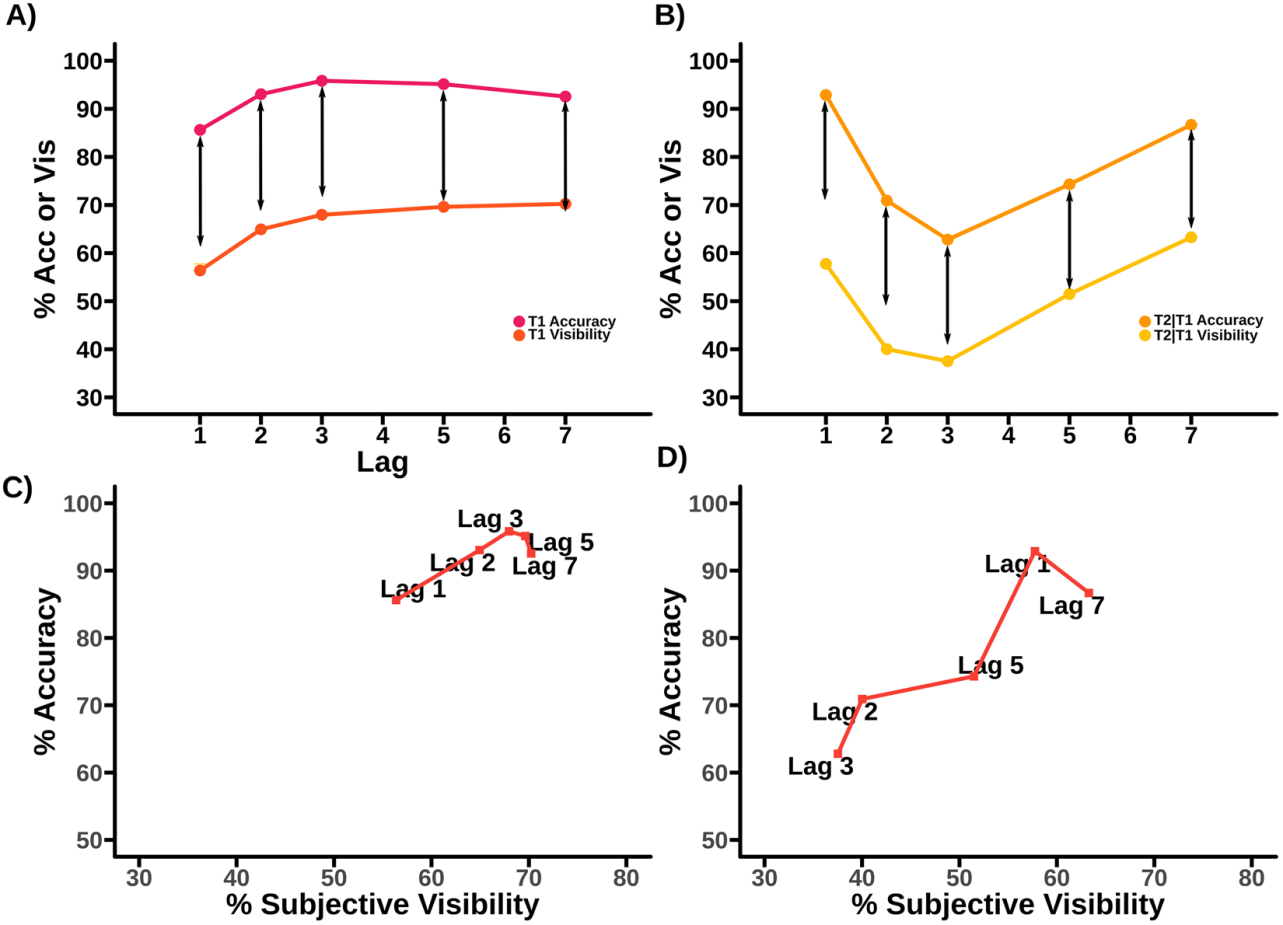
Behaviour of replication (pure letters in digits) data, comparing accuracy and subjective visibility across lags in the attentional blink. (**A**) A comparison of report accuracy and visibility ratings for T1. (**B**) A comparison of report accuracy and visibility ratings for T2. (**C**) A state-trace plot comparing accuracy and visibility for T1. (**D**) A state-trace plot comparing accuracy and visibility for T2. What we show as T2|T1 visibility is the visibility rating of T2 on all trials in which T1 was correctly reported. Note that compared to the analysis in^11^, T2 visibility shows a level of Lag 1 sparing. This dataset also measures visibility of the first target, which was not collected in the (colour-marked AB) study of^11^. Importantly, however, the basic dissociation of report accuracy and subjective visibility at short lags that underlies our hypothesis is qualitatively present for T2; see panel (B). For example, Lag 1 sparing is substantially higher for report accuracy than subjective visibility relative to other lags. This is illustrated by the black arrows, which indicate a constant distance for each graph. This can also be seen by noticing that, for T2 report accuracy, Lag 1 is considerably higher than Lag 7, while for subjective visibility it is marginally lower. Notice that the T1 curves do not seem to show the dissociation at early lags between report accuracy and subjective visibility that we see for T2. In particular, the differences in vertical distance across lag that are present in panel (A) may just be a facet of the small dip in T1 accuracy at later lags, a feature that we have not observed previously and which may just reflect “sampling error”.

One critiscism of an analysis based on the colour-marked data we present in Fig. 1 is that the the very substantial differences in report accuracy and subjective visibility at Lag 1 may be due to the use of a colour-marked T1. Previous experiments that have examined subjective report in the attentional blink often find some degree of sparing of subjective visibility at lag 1 (see, for example^8,24^), which is not observed in the colour-marked T1 data. In light of this, we propose a replication without a colour marked T1, giving a pure letters-in-digits paradigm. Details of the experimental procedure will be given in our materials and methods section, but the behavioural results can be seen in Fig. 6, and interestingly, we do see sparing for subjective visibility at Lag 1, although we will still be able to show the dissociation between report accuracy and visibility at Lag 1 that is central to our argument.

We also need to buttress ourselves against the possibility that we are observing a dissociation between report accuracy and subjective experience for reasons that do not entail the sight-blind recall effect we are considering. This might occur if there is a different mechanism modulating visibility at Lag 1, than at other data points. This is a very pertinent concern, since the Lag 1 data-point is often argued to be unique in respect of attentional blink lags; it is, for example, by far the most vulnerable to order errors^23^, or integration of both targets into one perceptual episode^25^. We take two routes to addressing this potential concern. Firstly, and most directly, we show that with the removal of the Lag 1 data-point in the replication (pure letters-in-digits) experiment just discussed, the effect still remains non-monotonic.

Secondly, contrary to a temporal integration explanation, a clear prediction of our proposal is that “if the individual P3s for two items are above the (conscious awareness) threshold, then the second item cannot be experienced until the P3 for the first one falls below threshold”. As a result, the visibility (relative to accuracy) for T1 should remain intact at Lag 1 compared to other lags, since it will be experienced to completion, or, in other words, the co-active T2 cannot interrupt the ongoing experience of T1. According to a temporal integration account, visibility of T1 should be impaired at Lag 1, since integration fundamentally suggests a T1-T2 “composite” is constructed, which would surely imply an impact of T2 onto T1. In contrast, we predict that T1 is isolated from the interference of a proximal T2. To address this concern, we propose a state-trace analysis of the T1 data of the replication (letters-in-digits) experiment. This has several advantages. First, it allows us to robustly examine whether visibility is changing differently with respect to accuracy across lags, when compared to our first (colour-marked) experiment. Second, a monotonic finding for T1 in the replication experiment would provide evidence directly against target integration.

One further analysis we perform is to examine report accuracy when participants indicate an absence of subjective visibility at Lags 1 and 3. This is a key analysis for the idea of sight-blind recall. That is, being able to show above chance report accuracy for T2, when participants select the bottom subjective visibility bin, i.e. nothing seen, suggests recall without experience. Showing that this phenomenon is larger at lag-1 than lag-3 further supports our position that co-activation (although not co-experience) of T1 and T2 particularly drives the dissociation of visibility from report accuracy. A preliminary version of this analysis was reported in the Supplementary Material of^11^. To maximise the available data for this analysis, we perform it on the second set of data from^11^, which sampled fewer lags with more trials, compared to the first set of data from^11^, which we have examined thus far in this paper. Focusing on this higher-powered data set enabled us to more robustly measure this effect.

## Materials and methods

### Original colour-marked RSVP data

#### Ethics

All experiments were performed in accordance with the relevant guidelines and regulations. The study was approved by the Psychology Research Ethics Committee at the University of Cambridge, UK and participants provided informed, written consent.

#### Data

Our set of data is a behavioural attentional blink dataset previously presented in^11^. Full details of the experimental procedure is given in the original paper, we summarize this here for clarity. Data was collected for two experiments, a behavioural set that sampled a large number of lags over fewer trials per lag (Experiment 1), and an electrophysiological set that additionally collected EEG data, and sampled fewer lags (Experiment 2).

Targets were uppercase letters and distractors were single digits, each trial contained one or two targets - T1 occurred on every trial and was always presented in red, and T2 (if it occurred) was presented in white. Targets could be any one of 21 letters, with 5 letters excluded because of similarity to numbers. Each RSVP stream contained 15 items. T1 randomly appeared as the fourth, fifth or sixth item in the RSVP stream. Stimulus Onset Asynchrony (SOA), the amount of time between the onset of each stimulus, was 90 ms. At the end of each RSVP stream, participants were asked to rate the subjective visibility of T2 using a 6 point self-report scale. The numbers 1 2 3 4 5 6 were presented in a horizontal line on the screen, with the description “not seen” presented beneath the number 1 and the description “maximal visibility” presented beneath the number 6. Participants then reported the identity of T1 and T2 (even if a second target did not occur). Participants were required to guess if they were unsure of the target identities. In Experiment 1, T2 appeared at lags 1, 2, 3, 4, 6, 8, or not at all with equal frequency. Results of this experiment for 18 participants were presented in Fig. 1. In Experiment 2, targets appeared at Lag 1 (40% of trials), Lag 3 (40% of trials), Lag 6 (10% of trials) and not at all (10% of trials). Experiment 1 deliberately sampled a large number of lags in order to examine the relationship between T2 accuracy and subjective visibility across the entire AB curve, while Experiment 2 sampled fewer in order to facilitate the creation of robust EEG data. Note that in contrast to the original study, for our state-trace analysis of second targets (T2s), we only include trials in which T1 is present and T1 and T2 are reported in the correct order in order to avoid order errors as a confound. This applies for both our accuracy and visibility ratings.

#### NImplementation specifics

##### Setting the prior

We set the prior of our Bayesian analysis from prior literature, specifically based on the results from^24^. This paper presents both a classic attentional blink with lag 1 sparing of report accuracy, and a similar “experiential” blink of subjective report in which lag 1 is spared a great deal less. Due to the well-established evidence for the pattern of behaviour in the attentional blink, we encoded strong expectations of behaviour, including lag 1 sparing, of the report accuracy in our data. Comparatively, the evidence for the behaviour of subjective report during the blink is less well established, so we refrained from imposing such strong constraints about it, particularly at the important lag 1 data point. We also recognise some uncertainty about the deepest point in the attentional blink: given the SOA of 90 ms, we could reasonably expect either of lags 2 or 3 to be the deepest point in the blink. We therefore set our prior to be consistent with several potential deepest points. Finally, Lag 8 is a serial position outlier (A common finding in attentional blink experiments is that a last lag that is a serial position outlier, e.g. if there is no Lag 7 and most lags in the experiment are short, participants will come to learn this regularity and optimize the allocation of attentional resources to short lags, causing lag 8 performance to be relatively low across the experiment.) in our experiment and was therefore removed from our analysis. These considerations resulted in a uniform prior subject to the following constraints across our data: for report accuracy, Lags 1, 4 and 6 would be held to be larger than Lags 2 and 3, with Lag 1 additionally being held to also be larger than Lag 4. For subjective report, Lag 6 would be held to be higher than Lag 4, Lag 4 higher than Lag 3, and Lag 3 higher than Lag 2. The validity of these constraints, as determined by our empirical priors method discussed in the Supplementary Material Section A was strong, but not completely homogenous. We therefore applied our method of empirical priors to reduce them to a set with a better fit. After application of our method, our prior was still uniform, subject to constraints as follows: For report accuracy, Lags 1 and 6 would be held to be larger than lags 2 and 3, and Lag 1 additionally would be held to be larger than Lag 4. The constraints for subjective report remained unchanged.

##### Distribution of data

The state-trace method we are applying, based on the work of^21,22^, assumes a binomial distribution of the data. This is suitable for our accuracy data, which is a dichotomous variable, but not for our visibility scale that forms a multinomial distribution over 6 values. Consequently, we grouped our visibility results into two bins, a high visibility bin and a low visibility bin. To decide the fairest way of applying this split, we calculated the grouped bayes factor comparing the validity of the constraints for each possible method of splitting the data, for both the full and empirically determined prior. The results (see Supplementary Material Section C) clearly show that the “best” split is that of assigning the top 50% of visibility ratings to the high visibility bin and the bottom 50% to the low visibility bin.

### Replication pure letters-in-digits RSVP Data

#### Ethics

All experiments were performed in accordance with the relevant guidelines and regulations. The study was approved by the Faculty of Sciences Ethics Committee at the University of Kent, UK and participants provided informed, written consent.

#### Data

Our data is a set previously presented in^33^, collected by Ellis Luise Gootjes-Dreesbach as part of her doctoral research at the University of Kent. 12 young adults took part in this study, aged 18–30 with a mean age of 21.83 years. Targets were upper case letter and distractors single digits. Targets could be any one of 21 letters, with 5 letters excluded because of similarity to numbers. Each trial contained two targets, with no colour marking for either target. Each RSVP steam contained 20 items. T1 randomly appeared as the 7th, 8th or 9th item in the steam. T2 was pseudorandomly presented at Lags 1, 2, 3, 5 or 7, ensuring an equal number of trials in each condition. Stimulus Onset Asynchrony (SOA), the amount of time between the onset of each stimulus, was 83 ms. At the end of the stream, participants were asked to respond (via the keyboard) to four questions about the visibiltiy and identity of T1 and T2. The query for target visibility (‘On a scale of 1–6, please indicate how well you saw the first [second] letter') was paired with an ASCII representation of a 6-point scale with the low end labelled as “not seen” and the high end labelled “maximal visibility”. Target identity was queried by asking “What was the first [second] letter you saw? If you are not sure, give your best guess”. We analysed all trials whatever the report order. The whole experiment consisted of 4 blocks of 45 trials, each randomised with respect to lag and T1 position.

#### Implementation specifics

##### Setting the prior

This experiment sampled slightly different lags to the original colour-marked experiment, but we attempted to replicate the constraints used in the previous experiments as closely as possible for the analysis of T2. Specifically, we substituted all constraints in the previous experiment, with Lag 5 replacing Lag 4, and Lag 7 replacing Lag 6. For T1, lacking any precedent in the literature for the behaviour of T1 visibility, we placed no constraints on the possible orderings of our data. For this replication experiment, in order that constraints did not change from those in the original data set, we did not make use of our method of deriving empirical constriants.

##### Distribution of data

To provide the fairest comparison to our original (colour-marked) analysis, we maintained the previous split of visibility ratings into high and low bins.

## Results

### Original colour-marked data

#### State-trace results (T2)

**Figure 7:**
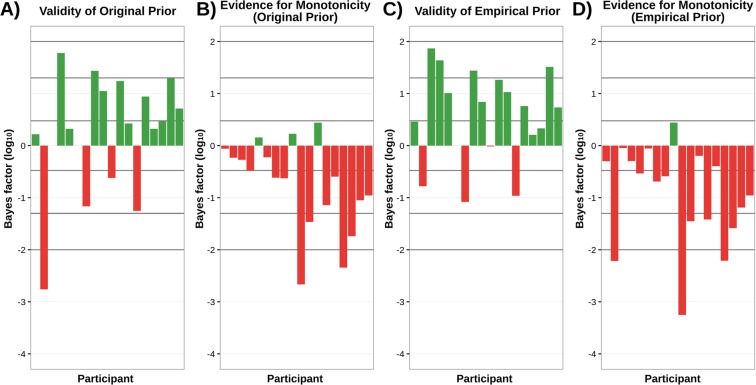
*Log*_10_ Bayes factors for each participant across 4 different tests, for T2 in the original (colour-marked T1) experiment. Note that participants are in the same order in all graphs to facilitate comparison. Lines overlaying the figure correspond to bayes factors of $\frac{1}{10000}$, $\frac{1}{1000}$, $\frac{1}{100}$, $\frac{1}{20}$, $\frac{1}{3}$, 3, 20, 100, and 1000 respectively. (**A**) Evidence for validity of the prior by participant for the original prior based on^24^. (**B**) Evidence for monotonicity (positive) vs non-monotonicity (negative) by participant for the original prior. (**C**) Evidence for validity of the empirically derived prior. (**D**) Evidence for monotonicity (positive) vs non-monotonicity (negative) by participant for empirically derived prior.

Figure 7(A) shows validity for each participant for the original set of prior constraints derived from^24^. At the group level, the evidence is strongly in favour of the constraints fitting the data with grouped (not log) *BF*_*D*/*N*(*D*)_ = 1.22 × 10^9^. However, we note that while the group validity is strong, four participants show the opposite pattern. Figure 7(B) shows the respective non-monotonicity for this set of constraints. Results are strongly and almost homogenously in favour of the non-monotonic model, with grouped (not log) *BF*_(*M*/*NM*)|*D*_ = 2.25 × 10^−14^.

Figure 7(C) shows validity for each participant for the set of prior constraints derived from the original using our empirical prior method. At the group level, the evidence is strongly in favour of the constraints fitting the data, with grouped (not log) *BF*_*D*/*N*(*D*)_ = 1.07 × 10^13^. However, we note that while the group validity is strong, there remains some variability across participants, though this situation has noticeably improved compared to 7(A). Figure 7(D) shows the respective non-monotonicity for this set of prior constraints. Results here are strongly and almost completely homogenously in favour of the non-monotonic model, with grouped (not log) *BF*_(*M*/*NM*)|*D*_ = 1.17 × 10^−17^.

### Replication letters-in-digits data

#### T2

**Figure 8:**
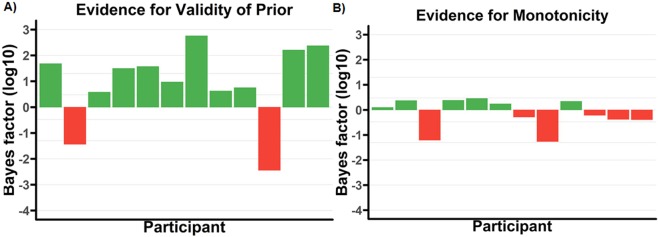
*Log*_10_ Bayes factors for each participant for monotonicity and validity of constraints for T2 in the replication (pure letters-in-digits) experiment. Note that participants are in the same order in all graphs to facilitate comparison. Lines overlaying the figure correspond to bayes factors of $\frac{1}{10000}$, $\frac{1}{1000}$, $\frac{1}{100}$, $\frac{1}{20}$, $\frac{1}{3}$, 3, 20, 100, and 1000 respectively. (**A**) Evidence for validity of the prior adapted from the original (colour-marked T1) analysis. (**B**) Evidence for monotonicity (positive) vs non-monotonicity (negative) by participant for this prior. Although the effect here is not as strong as it is for the original (colour-marked T1) experiment, the data does not exhibit the pattern in which the grouped Bayes Factor becomes a problematic measure, which arises, for example, if there is a single outlier subject driving the effect.

Figure 8(A) shows validity for each participant for the prior adapted from the original colour-marked T1 data analysis. At the group level, the evidence is strongly in favour of the constraints fitting the data with grouped (not log) *BF*_*D*/*N*(*D*)_ = 1.46 × 10^11^. Figure 8(B) shows the respective non-monotonicity for this set of constraints. Results are in favour of the non-monotonic model, with grouped (not log) *BF*_(*M*/*NM*)|*D*_ = 1.14 × 10^−2^.

#### T2 No Lag 1

**Figure 9:**
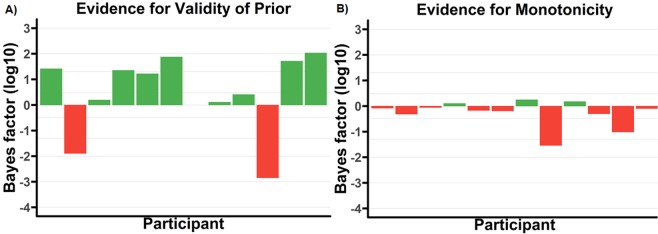
*Log*_10_ Bayes factors for each participant for monotonicity and validity of constraints for T2 in the replication (pure letters-in-digits) experiment with no Lag 1. Note that participants are in the same order in all graphs to facilitate comparison. Lines overlaying the figure correspond to bayes factors of $\frac{1}{10000}$, $\frac{1}{1000}$, $\frac{1}{100}$, $\frac{1}{20}$, $\frac{1}{3}$, 3, 20, 100, and 1000 respectively. (**A**) Evidence for validity of the prior from the (colour-marked T1) analysis. (**B**) Evidence for monotonicity (positive) vs non-monotonicity (negative) by participant for this prior.

Figure 9(A) shows validity for each participant for the prior adapted from the original colour-marked T1 data analysis, with Lag 1 removed. At the group level, the evidence is strongly in favour of the constraints fitting the data with grouped (not log) *BF*_*D*/*N*(*D*)_ = 2.5 × 10^9^. Figure 9(B) shows the respective non-monotonicity for this set of constraints. Results are in favour of the non-monotonic model, with grouped (not log) *BF*_(*M*/*NM*)|*D*_ = 5.75 × 10^−4^.

#### T1

**Figure 10:**
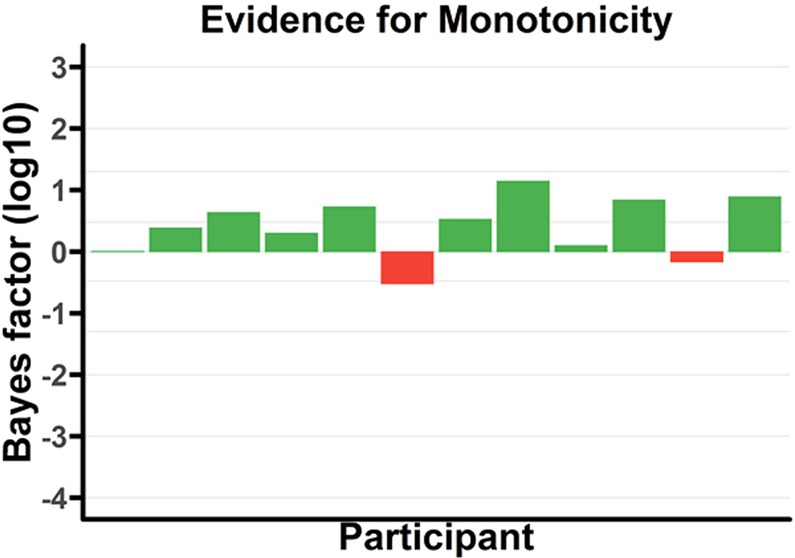
*Log*_10_ Bayes factors for each participant for monotonicity for T1 in the replication (pure letters-in-digits) experiment. Note that participants are in the same order in all graphs to facilitate comparison. Lines overlaying the figure correspond to bayes factors of $\frac{1}{10000}$, $\frac{1}{1000}$, $\frac{1}{100}$, $\frac{1}{20}$, $\frac{1}{3}$, 3, 20, 100, and 1000.

Figure 10 shows the respective non-monotonicity test for T1. Results are in favour of the monotonic model, with grouped (not log) *BF*_(*M*/*NM*)|*D*_ = 7.36 × 10^4^.

### Simultaneous type/serial token model results

**Figure 11:**
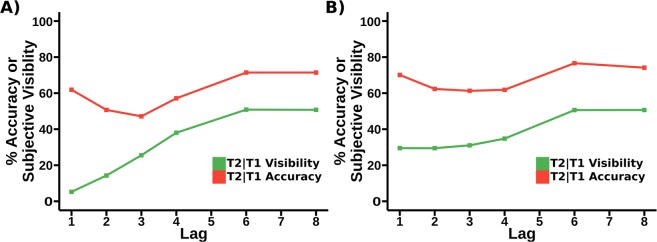
(**A**) Accuracy and subjective visibility by lag for the STST model. (**B**) T2|T1 Accuracy and T2 subjective visibility by lag for the data from^11^, i.e. the original (colour-marked) task. Note that these results have appeared in a different figure (Fig. 1(A)) above, but we present them reformatted here to better facilitate a comparison. Importantly, as previously discussed, neither the function or the structure of the STST model, as given in^10^ were changed when generating this fit.

Our first comparison is the behavioural results of the STST model and those from^11^; see Fig. 11. Note the qualitative similarity in behaviour. Such a high similarity between empirical and model findings is rare without a fitting of model parameters to the data.

**Figure 12:**
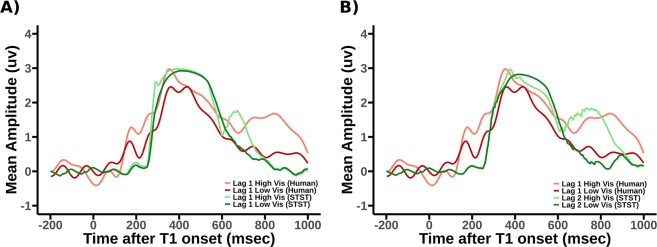
A comparison, for both high and low T2 visibility, given correctly reported T1, of the human ERPs from the original colour-marked T1 data analysis^11^. (**A**) Lag 1 Human ERPs vs Lag 1 STST virtual ERPs. (**B**) Lag 1 Human ERPs vs Lag 2 STST virtual ERPs. Importantly, as previously discussed, neither the function or the structure of the STST model, as given in^10^ were changed when generating the virtual P3s. Note that the human ERPs presented are slightly different to those from^11^, as ours exclude order errors to be consistent with previous sections.

We also compared the human ERPs with the virtual ERPs generated by the STST model, see Fig. 12. For full details on how these are obtained, see the Supplementary Information. We present two sets of model ERPs, comparing each of them to the same human ERPs, i.e. Lag 1. Panel A) compares to model Lag 1 and B) to model Lag 2. It should be clear from this that there are features of both the models Lag 1 and Lag 2 that are similar to the human Lag 1. This is perhaps not surprising and suggests a fixed offset timing difference between model and human data. Additionally, there are further reasons why it is unrealistic to expect a more perfect fit between simulations and empirical findings. Firstly, the task modelled by STST does not have a colour marked T1, which is likely to explain why the transient around 200 ms in the human data is not replicated by STST. Secondly, we are comparing scalp EEG directly to model deflections, without recourse to a forward (lead field) model of how brain sources are projected into sensor space. Critically though, the key property that a clear conscious percept of T2 (i.e. the high visibility condition) coincides with a longer P3 is qualitatively present in both sets of virtual ERPs. This pattern resonates with the notion that conscious perception imposes a seriality constraint that is not required for encoding into working memory. Some further results are available in Supplementary Section E, where we compare human and virtual ERPs at later lags.

**Figure 13:**
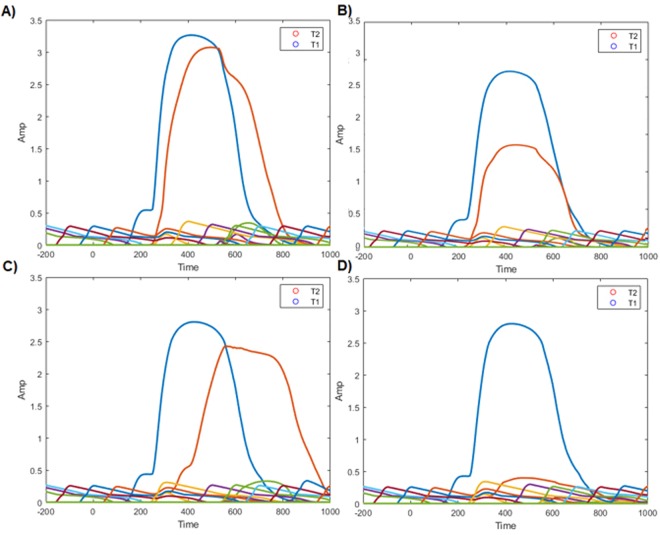
Activation traces by target for virtual data presented in Fig. 12, split up by visibility and lag. Unlabelled activation traces are from distractors. Each one of these activation traces corresponds to the sum of the excitatory post synaptic potential of the neurons on the 3rd, 4th, 6th and 8th layers of the neural-STST model, corresponding to the item layer, the task filtered layer, the binder gates and the token gates. This is illustrated in Fig. 4. The ‘full' activation traces that are presented in Fig. 12 are generated from the sum of each of these individual traces at each timepoint, subject to the seriality of experience we have discussed previously; when one target is being experienced, the activation trace of the other target (or indeed, distractors) makes no contribution to the grand activation trace. (**A**) individually depicted activation traces from the SESE model for each target, for high visibility targets at Lag 1. (**B**) individually depicted activation traces from the SESE model for each target, for low visibility T2s at Lag 1. (**C**) individually depicted activation traces from the SESE model for each target, for high visibility targets at Lag 2. (**D**) individually depicted activation traces from the SESE model for each target, for low visibility T2s at Lag 2.

For illustrative purposes, we also present the activation traces for high and low visibility, for each of the T1, T2 and distractors seperately. We do this for each lag separately. This can be seen in Fig. 13(A,B) (Lag 1) and Fig. 13(C,D) (Lag 2). This clarifies how the Virtual ERPs in Fig. 12 emerge from the underlying STST activation traces. An STST virtual ERP, as presented in^31^, is a summation of the traces in a panel of Fig. 13, including the low amplitude responses to distractors, which contribute to the “rougher” contours of the Fig. 12 model time series compared to the Fig. 13 target time series. Critically, the experience read-out mechanism we are proposing here means that the T1 and T2 traces are not simply summed when they are co-active. Rather, the T2 trace only starts contributing to the virtual P3 once the T1 trace has fallen below the visibility threshold, as shown in Fig. 5. Accordingly, only the the back-end of the T2 trace in Fig. 13(A) contributes, almost none of it in Fig. 13(B) and a much larger proportion in Fig. 13(C).

### Report accuracy at minimal subjective visibility

To further justify the term sight-blind-recall, we directly investigated T2|T1 accuracy at the lowest level of subjective visibility. The question of interest is whether we can actually demonstrate that report accuracy is above chance when subjects report zero visibility of the T2. To this end, T2|T1 accuracy was calculated only on trials where participants selected a visibility rating of 1 (the lowest possible visibility rating, indicating ‘not seen'). For each lag, T2|T1 accuracy was compared with the degree of accuracy expected due to chance (4.76%, one out of 21 letters presented), using one-sample t-tests. In other words, we investigated whether T2|T1 accuracy was greater than 4.76%, at relevant lags. As discussed, this analysis was conducted for lags 1 and 3 in the (colour-marked T1) second experiment from^11^, as that is where the trial counts were sufficiently large to examine a specific subjective visibility (200 trials for each of those lags). As expected, accuracy was significantly greater than chance, despite participants indicating that the subjective visibility of the target was nil (lag 1: *μ* = 37.98%, *σ* = 25.25%, t(1,17), p < 0.001, d = 1.3156), (lag 3: *μ* = 15.03%, *σ* = 12.5%, t(1,17), p = 0.0014, d = 0.8214). We also examined the hypothesis that at minimum visibility report accuracy at lag 1 was greater than report accuracy at lag 3. We found evidence for this hypothesis, (lag 1 > lag 3, t(1,17) = 5.2033, p < 0.001, d = 1.2264).

## Discussion

### Monotonicity verseus non-monotonicity

Our state-trace analysis, comparing the measures of accuracy and subjective experience in the attentional blink, found strong evidence for a non-monotonic model of the relationship between these two measures at both the individual participant and group level. This was further supported by the methods developed as part of our own contributions to the current state-trace methodology. We would argue that our empirical priors approach identifies a more accurate set of results across the data, however it is encouraging that our results are similar both with and without our empirical priors.

Previous literature^21^ has advocated the use of both the Grouped Bayes Factor (GBF) that we have calculated, as well as an Aggregated Bayes Factor (ABF) to confirm the homogeneity of the results, something we have not done. There seems little need to apply the ABF, since our data shows substantial homogeneity in both contrasts for which it is tested: for example, considering our main state-trace finding for our original colour-marked T1 data set, only three participants demonstrate even incidental evidence for a monotonic model (cf. Fig. 7B) with the original prior, and only one with the empirical prior (cf. Fig. 7D). Additionally, we note that the ABF cannot be used to confirm homogeneity, only identify heterogeneity.

There is one potential exception to this, Fig. 8B). In this instance, ignoring the absolute quantity of the effect, exactly half the participants show one Bayes factor direction, and half the other. This is heterogeneous in nature, which, as we have discussed, may be a problem case for the GBF. However, in this instance, we do not believe that we need to be overly concerned. The dangerous case of heterogeneous results in respect of the GBF is that it can potentially lead to a misleading summary of the overall effect. However, that is not the case in Fig. 8B). While it is true that we have a substantial number of participants supporting both monotonic and non-monotonic directions, the only non-incidental Bayes Factors we have provide evidence for non-monotonicity. In this case, the most natural interpretation of the data is non-monotonicity, which supports the calculated GBF.

One aspect of our analysis that is notable is the lack of a trace factor. However, the introduction of a trace factor is only required in the case in which there are only two levels of the dimension factor; in other cases, the introduction of a trace factor is a convenience designed to sweep out the behaviour of a system. In our case, we have 5 levels of our dimension factor, which is very close to, or exceeds the combined total trace × dimension factors in other state-trace experiments^18,28,34^.

### Working memory encoding without subjective experience

Our results suggest some kind of dissociation between working memory encoding and subjective report. Despite this, we have only demonstrated that a dissociation exists and have not definitively characterised it: we would claim that our findings are indicative of a particular relationship of dependency between working memory encoding and conscious perception, but no more than that. However, our results do not exist in a vacuum. It is clear that the dissociation we observe is a phenomenon of very short lags. In particular, it is largest at Lag 1. For example, in the original (colour-marked T1) study, the series of interactions performed in^11^, in which lags were systematically excluded, suggest a strong dissociation at Lag 1, with weakening dissociations from Lag 2 to Lag 3 and nothing at higher lags, additionally, the state-trace analysis performed here on that same data showed non-monotonicity when all lags were included, but the removal of Lag 1 from the state-trace analysis nullified that effect, see Supplementary Material Section B for details of this analysis. Furthermore, the state-trace analysis we perform here on the replication (letters-in-digits) data set shows non-monotonic patterns with all lags in and when Lag 1 is excluded, but the effect is lost when further lags are excluded.

A dissociation restricted to just very early lags, and particularly Lag 1, raises the possibility, but no more than that, of working memory encoding being a necessary, but not sufficient, condition for conscious perception (although, the existence of phenomenological awareness would mean WM encoding was also not necessary for conscious perception). This is because it is at these lags that the activation of T1 and T2 is most strongly simultaneous. Thus, we can say that it is specifically when T1 and T2 are active together that T2 is encoded into WM, with a weakened, or absent, perceptual experience, suggesting a capacity to encode T1 and T2, while the T2 conscious percept is impaired. In addition, our finding in subsection “Report accuracy at minimal subjective visibility”, that there is above chance report accuracy when participants report zero visibility, an effect that is substantially stronger at Lag 1 than Lag 3, provides probably the most direct evidence that on some trials encoding into WM can occur without visibility.

We also view the P3s we have observed in the original (colour-marked-T1) experiment as consistent with this interpretation although certainly not definitive verification of it. For example, in Fig. 12, it is clear that the Lag 1 High Vis (human) is considerably longer than the Lag 1 Low Vis (human). Additionally, in^11^, Fig. 8 compares the ERPs for T2 correct with T2 high visibility (compare the green traces in panels A and F), again the high visibility T2 has a substantially extended P3. This seems to suggest that consciously seeing the T2 dramatically extends the P3, while the curtailed P3 when T2 is just correct, but not necessarily vividly seen, might be considered indicative of a T2 being encoded, with little, if any, conscious experience.

This profile of findings could suggest a phenomenon called “sight-blind recall”, however, further empirical support from the RSVP domain and beyond is required to fully justify this interpretation. In particular, the critical demonstration would be that when T2 is correctly reported but given a zero visibility response, the lag 1 P3 is the same as that for a T1 alone. We do not though have sufficient trials in our ERP experiment to reliably construct this average. This, then, is a key test that needs to be performed.

Importantly, this purported sight-blind recall is different from more familiar notions of preconscious processing, such as subliminal priming, implicit perceptual learning as well as related findings demonstrated with continuous flash suppression^15^ and phenomena such as blindsight^17^, or episodic face recognition^18^. These experiments demonstrate only an indirect effect on a later test; in no case is the “invisible” stimulus that is not consciously perceived directly reportable. We would argue that these results are not strong enough to demonstrate the “sight-blind recall” that we have described, indicating instead influence without experience. In contrast to this, our results suggest the potential for free recall of a stimulus that has not been conscious perceived, a much stronger result that we would argue is far closer to constituting sufficient evidence for “sight-blind recall” and working memory encoding without conscious experience.

The decoupling of subjective visibility from report accuracy at early lags is particularly striking in our original (colour-marked-T1) data set, where there is no evidence of Lag 1 sparing for subjective visibility at all; see Fig. 11(B). However, it is important to realise that the decoupling effect we have identified is not dependent upon the complete absence of sparing for subjective visibility, and this is important, since other studies that collected subjective visibility, e.g.^8^ and^24^, did see lag-1 sparing for subjective visibility. Importantly, the replication (letters-in-digits) data set, indeed, has sparing of subjective-visibility; see Fig. 6. However, critically, this kick-up at early lags is, in relative terms, considerably smaller for visibility than for report accuracy. Accordingly, we are still able to demonstrate the state-trace non-monotonicity that is central to the argument in this paper, and, in fact, the interaction that was central to^11^ can also be demonstrated, see^33^.

These findings though raise the question of why different lag-1 subjective visibility patterns have been observed, i.e. why is it that the original (colour-marked-T1) data did not show lag-1 sparing for subjective visibility, but^24^ and our replication (letters-in-digits) data set did? Considering our data sets, one factor that surely impacts this is the T1 colour-mark in the original study. This, we believe, makes the T1 perceptually strong and, also, more easily distinguishable from the T2. Indeed, in this data set, T1 report accuracy is considerably higher than T2 report accuracy performance at all lags.

In contrast, the replication (letters-in-digits) study was a straight letters-in-digits task, with no colour marking. This may have caused the T2 to be more strongly perceived, since the T1 is not as strong as it is in the original (colour-marked-T1) study. It is less clear how to reconcile our findings with^24^, since they did have a colour-marked T1. However, their colour-marking may not have been as salient as ours: cyan in theirs versus red in ours. This could potentially mean that there is also increased relative strength for T2s in their experiment, increasing its visibility. A definitive answer to these inconsistencies, though, awaits further empirical work.

Broadening out fron the attentional blink, there are several pieces of work that present findings consistent with our results. Firstly, evidence of working memory maintenance without conscious awareness^35^ sits very nicely with our results, and this is even more the case for such a demonstration with the attentional blink^36^. If we have indeed found a case in which working memory representations can be formed, without awareness of their formation then we would have identified an explanation for how items could enter working memory without being experienced, which then could be maintained without experience. Our results may help explain how these pre-conscious working memory traces arise by giving them a mechanism through which they can be encoded without conscious experience^37^ also present experimental conditions in which they are able to use metacontrast masking to vary the subjective report of consciousness, while stimulus discriminability is maintained. Further, the authors find that as SOA decreases (down to around 50 ms, at which point the effect reverses) shorter SOAs result in lower subjective experience, consistent with our finding that subjective experience drops as T1 and T2 become closer^37^. is a landmark study; our results, though, move beyond their work by applying state-trace analysis rather than single dissociations, and by considering identification with free recall, rather than two alternative forced choice decisions. In this sense, our objective behaviour relies upon a significantly more complex cognitive process.

Taking our results along with those from^1,3,4^ that indicate some degree of perception without reportability, it may be tempting to conclude that working memory encoding and perception are highly correlated but mutually dissociable processes. However, all of the studies above provide their evidence in the form of the single dissociations. Further state-trace analysis could provide additional evidence for the dual question to that studied in this paper.

From a theoretical point of view, it is interesting that perception is most taxed at Lag 1. As we have discussed^11^, note that this pattern of behaviour is consistent with a model of the attentional/experiential blink in which stimuli are consciously perceived in a serial manner, but encoded in a simultaneous manner. This is discussed in further detail below.

### Integrated percepts

One potential criticism of our results is that the low subjective experience at Lag 1 is caused by the rather unique nature of the Lag 1 data point. Lag 1 is the only data point without any intervening distractors, and is, notably, by far the most vulnerable point to order errors^23^, or integration of both targets into one perceptual episode^25^. In this case, the poor report of subjective experience of T2 might be confounded by the presence of T1. Participants might report poor T2 visibility not because T2 was not vividly experienced, but because the experience of T1 in the same perceptual episode causes confusion. This issue was discussed at length in^11^, but we return to the point, since it remains an important potential confound that is worth revisiting in the light of the new findings being presented in this paper. We additionally note that there are an unusually small number of putative integrated percepts in the experiment of^11^. The colour marking of T1 in this experiment reduced the classical indicator of integrated percepts, order errors, from 30% in classic letters/digits tasks^38^ to approximately 10% in the task from^11^. Further, we note that the pattern of behaviour we see at Lag 1, with low subjective experience and high accuracy is also visible to a lesser extent at lags 2 and 3, in which there are intervening distractors.

Another important point that stands against an integrated percepts explanation is the evidence that the reduction in relative subjective visibility can also be observed at Lag 2, and perhaps also weakly at Lag 3. The interaction analysis in^11^ showed this, and the state-trace analysis we performed in this paper, suggested a non-monotonic pattern was still found in the replication (letters-in-digits) task when Lag 1 was removed. The integration argument is though classically ascribed specifically to Lag 1 and not later lags, in which there are intervening distractors. A further reason for believing that perceptual integration is unlikely to explain our findings is that it seems T1 is immune to the decoupling of report accuracy and subjective visibility, a point we discuss next.

### Target specificity of decoupling

Importantly, the replication (letters-in-digits) data set that we analyse in this paper strengthens the specificity of the argument we are able to make. This further data set has enabled us to, firstly, replicate the decoupling between report accuracy and subjective visibility for T2. This was done with the state-trace analysis of T2 reported in subsection “Replication (letters-in-digits) Data” of section “Results”. In addition^33^, reports the classic T2 interaction between Report Measure (report accuracy vs subjective visibility) and Lag for the letters-in-digits data set, which we reported in^11^ for the original (colour-marked T1) data set.

Secondly, and perhaps most significantly, while subjective visibility ratings for T1 were not collected in the original (colour-marked T1) data set, the replication data set has that data point. As a result, we have been able to investigate whether there is a dissociation of report accuracy and subjective visibility for T1; and, importantly, there does not seem to be one^33^ failed to find an interaction between Report Measure (report accuracy vs subjective visibility) and Lag, and, in this paper, we identified a monotonic state-trace pattern for T1 in the replication data set; see subsection “Replication (letters-in-digits) Data” and Fig. 10.

The immunity of T1 to the report accuracy – subjective visibility dissociation suggests that the relationship between working memory encoding and conscious perception is unchanged across lags, and, notably, that co-activation of T1 with T2 (as occurs at very short lags) does not impair the conscious experience of T1, in the way it does T2. This finding is wholly consistent with the serial experience interpretation we are arguing for in this paper. That is, at very short lags, particularly Lag 1, T1 typically starts being perceived before T2 does, conferring it occupancy of the exclusive “focus of conscious experience”, and the, late coming, T2 is excluded. This manifests in a, relative (to report accuracy), loss of visibility for T2, but not for T1, which is what we observe. In other words, the T1 claims “the brain’s experiencer” before T2 arrives, and holds it until T2 has decayed, but there is no such exclusivity to the encoding into working memory.

This T1 immunity to the report accuracy – visibility dissociation also stands against a perceptual/event integration interpretation. This is because, at its very heart, event integration suggests a composite of T1 and T2 is experienced. But, if that were the case, one would surely expect any impairment in T2 visibility associated with that composite, to also impact T1, In other words, if one is going to argue that T2 subjective visibility being low at Lag 1 is due to a confused “joint” binding, why would that decoupling of subjective visibility and report accuracy not also impact T1?

### Simultaneous type/serial token model

There is no certainty with regard to an explanation of data such as we are presenting in this paper, but a computational account is as good a demonstration as one can have that a group of theoretical positions are consistent with each other, since a computational model has to run and generate this range of phenomena. Thus, we would argue that the STST computational account and the extension of it in the current paper is the demonstration that the theoretical positions we are taking are reconcilable. In particular, this shows that the subjective visibility findings we have named the Experiential Blink are reconcilable with the STST computation model, in particular, additions to the simultaneous type/serial token (STST) model of temporal attention allow it to index subjective experience as well as report accuracy, with the goal of providing a model that can explore the dissociations we discuss in this paper. In order to verify this model, we compared its predictions with the human data from^11^. The first comparison we made is between the behavioural results, specifically, we compare the respective report accuracies and subjective visibilities predicted by the SESE model to those from the human data. The results from this can be seen in Fig. 11(A,B). Overall, there is a strong similarity between the two. One notable difference is that the SESE model is simulating a slightly more difficult task than the human data – report accuracy lower by around 10%. Perhaps because of this, the SESE model also demonstrates a more marked downturn in subjective report at earlier lags than the human data.

We also compared the virtual ERPs generated by the SESE model with the human ERP data. The most significant difference between the two is the respective late dynamics of SESE compared to the human data, with the SESE data ERPs showing differences to the human data from approximately 600 ms onward. Despite this, there is still a strong qualitative fit between the SESE data and the human data. It is important to note that we have taken the STST model exactly as it was formulated over 10 years ago, i.e. in^10^. Most notably, we have not refitted the parameters of the model in order to improve the match to the experimental data presented in this paper. This surely means that the match between model and experimental data is not going to be quantitatively perfect. In this respect, it is perhaps only reasonable to just expect a qualitative match between model and experimental results. In this context, the quality of match to the empirical data is, we would argue, impressive. Most importantly, the simulations we have run with SESE have provided a proof of principle that the explanation presented in Fig. 5 for why report accuracy and subjective visibility diverge is tenable. This explanation rests on the concept that encoding into working memory can proceed in parallel, but conscious perception cannot, a concept which we have noted suggests a theory called simultaneous encoding/serial experience. The natural electrophysiological correlate of this is a time-extended P3 when both T1 and T2 are consciously perceived, as opposed to just T1. This is what we observe in our data, and simulations in Fig. 12.

It is also important to observe that without a full investigation of the range of input strengths and parameter values within the STST family of models, the full range of patterns of data that can be embraced by the SESE model is not certain. For example, in its current configuration, the model generates very low visibility at lag-1 (see Fig. 11), which seems inconsistent with the observation that subjective visibility can exhibit sparing at lag-1, just substantially less than observed for report accuracy; see Fig. 6B). However, within the STST family of models, there may be a region of parameter settings that enable weak sparing for visibility at lag-1. In particular, the model is on something of a “knife-edge” at lag-1 and small changes in input strength and parameter settings can greatly change the model’s behaviour.

One possible way in which sparing could be obtained for visibility would be if the T1 activation trace were high amplitude but short in duration, only excluding perception of T2 for a short period and thereby enabling it to be seen relatively vividly. If this were accompanied by very weak activation traces for T2 during the blink, weak lag-1 sparing of visibility may be obtainable. In this respect, aspects of the eSTST model^23^ could be relevant, since they enable a more marked difference in dynamics between sparing and the blink. These aspects ensure that it is hard to reactivate the blaster (STST’s attentional enhancement) once a blink has been initiated, naturally leading to weak T2 activation traces at lags 2 and 3. This said, modelling sparing of visibility at lag-1 is likely, at the least, to require retuning of STST’s parameters, a step we have avoided to date.

A potentially far-reaching claim of the SESE model is that the generation of P3s is more involved than previously proposed (see^31^) for STST. We are not in a position to completely define this approach with full neural detail; that has to await further work. However, the new interpretation is required in order to be consistent with the results we present here and particularly in^11^. Specifically^11^, suggests that the P3 indexes conscious perception, not working memory encoding, so if we are proposing seriality of conscious perception, we have to propose seriality of the P3. Although a definitive mechanistic explanation awaits further modelling work, the intuition is that the activation traces currently generated by STST (which aggregate across a number of layers of the model) are precursors to the actual P3 and are earlier in the processing pathway. These activation traces feed into our “readout” mechanism, which is serial, excluding the second target from contributing to the P3 until the first has completed being experienced, i.e. has dropped below threshold.

Thus, we are imagining that the activation traces for T1 and T2 that the original STST model generate remain unchanged and can unfold in parallel, as they currently do at lag-1. Working memory encoding is still driven from these traces, but conscious experience is driven by the traces read-out, a notion that could be related to ideas of self-observation prominent in theories of conscious experience^39–41^. This readout enhancement can be considered speculative at this point. However, we include the idea here, since one purpose of theory is to provide strong claims that empirical work can attempt to disprove. This is a classic example of a scientific prediction that would be considered unlikely unless one subscribes to the theoretical position associated with the SESE theory. These are exactly the predictions that can carry the most evidence if experimentally investigated.

Indeed, it is central to scientific progress that testable predictions are made from models, in order that formalised theories can be disproved, the key to scientific progress from a Popperian perspective. In this spirit, the SESE model that we have presented in this paper makes two particularaly strong claims. The first being that that the P3 at lag-1 does not have the form of a double-amplitude single-target P3. Note, the vanilla STST, without readout-enhancement, does generate a double-amplitude P3 at lag-1, see Fig. 7 of^31^. Critically, it is important to rule out the possibility that the observed lag-1 P3 is reduced in amplitude because it is at ceiling. That is, the specific prediction is that the lag-1 P3 is a similar amplitude to a single-target P3 and the distribution of P3s observed is not skewed according to a ceiling effect. The second key prediction that the SESE P3 readout mechanism predicts is that the steady state visual evoked potential (SSVEP) weakens or even de-synchromises during the P3. This is because if one asserts that an ongoing P3 for a target excludes the activation trace for another target, it should also exclude or dampen the activation traces of distractors (which drive the steady state response). Clearly, the SSVEP is at least partially from generators substantially earlier in the processing pathway than those that might directly drive the P3. Nonetheless, some sort of reduction in the power of the SSVEP may be observable. Disproving the first of these predictions would be a major problem for the readout-enhanced STST theory. Finding evidence for the second would provide converging evidence for the theory.

### Seriality and STST

It is important to clarify the STST theory in the light of the findings and the serial experience ideas presented here. The following are key points to consider.

1. The original STST theory already makes a seriality assertion^10^. This, though, is a seriality over a longer time-frame than we are considering in this paper. That is, it proposes that the attentional blink has the role of delaying the start of a second episode, in order that all the bindings associated with a first episode can be completed before the next one starts. Thus, the seriality it focusses on is “across” the attentional blink, e.g. between a T1 and a T2 at, say, lag 5. As currently framed, it is focussed on working memory encoding, and does not explicitly speak to conscious experience.
2. The seriality considered in the current paper, is focussed on what happens when targets are very close together in time, e.g. at Lag 1. The original STST theory presented in^10^ incorporated the notion of a “joint encoding” at Lag 1, whereby both T1 and T2 can be encoded into WM, but with a loss of episodic information, e.g. order and conjunction properties. The Experiential blink and the experience read-out theory presented in this paper extends the “joint encoding” notion from the original STST model, by arguing that there can be “joint encodings”, but for T2 to be experienced, it has to be sufficiently strong that it can outlive the experiencing of T1. This is a new idea to the STST framework. The serialising considered here is specifically about conscious experience (the serialising of point 1. above is about working memory encoding), and it specifically occurs within a single episode, not across them.

## Conclusion

We have examined the evidence for a dissociation between working memory encoding and subjective report in the attentional blink, and developed our own additions to current state-trace methodology. Our data stands clearly for a dissociation between working memory encoding and subjective report, and examining the data shows that this is the result of an increase in accuracy and a decrease of subjective visibility at lags 1, 2 and 3. Overall, we may have found evidence for a case in which it is possible to encode a stimulus into working memory without consciously perceiving it, a phenomenon we call sight-blind recall; however, a good deal more evidence needs to be acquired before this claim can be made with confidence. The SESE model is consistent with findings from human participants, and the results of the state trace analysis of this current work. However, more work will be required to determine the further predictions that the SESE model makes, and the sparseness of literature with respect to the experiential blink will require further experimentation to validate the predictions presented in this paper and those that will emerge. In particular, although there are a number of competing explanations of the decoupling of report accuracy and subjective visibility we observe (see^11^ for a detailed consideration of many of these), evidence for the capacity to encode in parallel and experience in sequence is accumulating.

## Supplementary information

Supplementary Information.

## Data Availability

All of the code used in this project has been open sourced on Github, subject to an MIT liscence. See https://github.com/william-r-jones/StateTrace for the modified state-trace code, and https://github.com/william-r-jones/SESE for the modified STST model. All of the data used in this paper is also available alongside this code where possible, though some datasets (notably the EEG data) are too large for this to be possible and have instead been made available using the Dataverse Project. See https://dataverse.harvard.edu/dataset.xhtml?persistentId=doi%3A10.7910%2FDVN%2FU9DFFI.
