# Supplementary material for: Fleeting Perceptual Experience and the Possibility of Recalling Without Seeing
Source: Sci Rep. 2020 May 22;10:8540. doi: 10.1038/s41598-020-64843-2 (PMC7244573; doi:10.1038/s41598-020-64843-2)
Supplement: Supplementary file 1 — Supplementary Information. [file 41598_2020_64843_MOESM1_ESM.pdf]

Fleeting Perceptual Experience and the Possibility of Recalling Without Seeing

William Jones[a,1], **Hannah Pincham**[b], **Ellis Luise Gootjes-Dreesbach**[d], **and Howard Bowman**[a, c]

[a]Centre for Cognitive Neuroscience and Cognitive Systems, University of Kent, Canterbury, UK
[b]South Eastern Sydney Local Health District, NSW, Australia
[c]Department of Psychology, University of Birmingham, Birmingham, UK
[d]Point Estimate Limited, Ellesmere Port, Cheshire, UK

Supplementary Information

Supplementary Information

Section A - Extensions to the state-trace method

Previous state-trace analysis has generally been in a position to make strong statements about the ordinal relationships of the variables for which the measures of interest (e.g., accuracy and visibility) are calculated, allowing them to make strong statements with their priors. For example, in their experiment on short term memory[1] are able to a-priori assume in their data that accuracy in a change detection task is higher when participants have the opportunity to verbalise the first target than when they did not. In comparison, while we have strong expectations about some behaviours of the attentional blink such as lag 1 sparing in letters-in-digits tasks[2–4], the variability in, for example, depth of the blink between experiments, means we are not in a position to make such strong ordinal statements as these previous works. We therefore propose a data driven method that makes use of an orthogonal measure to the monotonicity contrast. This method takes two sets of a-priori "constraints" on the data, restrictions on potential orderings in the prior entered into Bayesian inference. These are an "irrevocable" set containing those constraints that no theorist would believe violable, and for which any evidence against can only be considered a measurement error - for example, we would expect lag 1 accuracy to be larger than lag 2 accuracy at the participant level in the letters-in-digits attentional blink - and a "free" set encoding those behaviours that we might expect to change between experiments – for example, the lowest point in the blink. Orderings of the dimension (or trace) factor that do not fit the constraints are considered a-priori to have a prior probability of 0, with all other orderings equally likely. Our method then removes constraints from the free set that do not fit the data on the basis of our orthogonal measure of validity. The result is a theoretically grounded, empirically derived set of constraints on the data.

This orthogonal measure is a dimension vs non-dimension factor, analogous to and intersecting with, the trace vs non-trace factor used in[1]. In the same way as this trace vs non-trace factor, this gives us a measure of how accurately the data conforms to a given set of ordering constraints across both the dimension and trace factors. We call this measure $BF_{(D\&T)/N(D\&T)}$, or when no trace factor is present such as in the main body of this paper, as $BF_{D/N(D)}$ in order to prevent confusion about the trace factor that does not exist in our analysis. In the case in which the trace factor has only one level (such as in our data), this measure is also equivalent to how well the data conforms to exclusively the dimension axis versus how well it does not. This measure specifically quantifies the ratio of evidence for the intersection of both the trace and dimension constraints versus all other points, thereby providing a measure of validity that the overall set of constraints we select fit our data.

In order to make use of this measure to derive a prior, we first pick a set of order constraints on the state and dimension axes from prior data, $C = \{c_1, \ldots, c_n\}$. This set of constraints should be the fullest set that can be reasonably expected to fit the data, but should not contain constraints that contradict one another. We then divide this set C into two subsets, those constraints in C for which violation can only constitute a measurement error (the irrevocable set), and those about which we might expect variation between experiments (the free set). We label these $E = \{e_1, \ldots, e_l\}$ and $F = \{f_1, \ldots, f_q\}$ respectively. Next, we introduce the concept of group validity for a given set of constraints, denoted GE. This is the product of $BF_{(D\&T)/N(D\&T)}$ across all our M participants for the set of constraints C, specifically:

$$GE(C) = \prod_{i=1}^{M} BF_{(D\&T)/N(D\&T)_i}$$

For each item in F, we denote the "leave one out" subset of constraints ($\overline{F}_j$) as:

$$\overline{F}_j = E \cup \{f_1, \ldots, f_{j-1}, f_{j+1}, \ldots, f_q\}$$

We then calculate $GE(\overline{F}_j)$ for all $j \in q$. For the largest evaluated $GE(\overline{F}_j)$ with $GE(\overline{F}_j) > GE(E \cup F)$, we then remove f_j from F. This procedure is repeated on the new F with f_j removed until there does not exist a set such that $GE(\overline{F}_j) > GE(E \cup F)$, or until $F = \{\}$. The resulting $E \cup F$ is the "empirical prior". We note that this method is very similar in its essence to the parametric empirical Bayes (PEB) method[5], however, we note that the specifics of our application allow us to solve the problem in a greatly simplified manner.

Our method is justified as follows. Firstly, it is clear that setting our empirical prior based on $BF_{(D\&T)/N(D\&T)}$ will, on its own, converge to a prior set of constraints that best fit the data. Secondly, since we are starting from the fullest (strictest) set of constraints that are theoretically grounded and pruning from this set, it is impossible for us to introduce spurious constraints that fit the data by chance, but are incompatible with our theoretical understanding. Equally, because we hold some constraints "irrevocable" we are protected from removing constraints that are highly likely a-priori, based on measurement errors. Finally, $BF_{(D\&T)/N(D\&T)}$ is an orthogonal measure to the $BF_{(M/MN)|/(D\&T)}$. Since $M|(D\&T) \cup NM|(D\&T) \subseteq D\&T$ (the union of the monotonic and non-monotonic orderings given some set of constraints is contained inside the set of all possible orderings given those constraints) the changes in the balance of probabilities between $M|(D\&T)$ and $NM|(D\&T)$ (calculated as $BF_{(M/MN)|/(D\&T)}$ have no effect on the respective probabilities of a given set of constraints $D\&T$ versus their complement $N(D\&T)$.

Section B - Lag 1 as a cause of non-monotonicity in the original colour-marked T1 task

In the main body of the paper, we find evidence for a strongly non-monotonic relationship between accuracy and subjective report in the original colour-marked T1 task. As noted in[6], this appears to be driven by differences in the behaviour at early lags, particularly Lag 1. Here, we attempt to quantify this effect by removing Lag 1 from the state-trace analysis, and examining how it changes. As well as removing the lag from the dataset, we must also adjust our constraints. The strongest performance was on the empirically derived constraints, so for this analysis we use these, minus any constraints on the lag 1 datapoint that are now no longer applicable. We find that, despite the fact that grouped (not log) evidence is almost completely unchanged ($BF_{D/N(D)} = 1.07 \times 10^{13}$ with lag 1, $BF_{D/N(D)} = 1.01 \times 10^{13}$ without), our grouped (not log) bayes factor changes from extremely strong evidence for non-monotonicity at $BF_{(M/NM)|D} = 1.17 \times 10^{-17}$, to no strong evidence either way . The results for each subject individually can be seen in figures 1 and 2. From this we conclude that Lag 1 is a strong driver of the effect of non-monotonicity that we see in our state-trace analysis of the original colour-marked T1 task. However, the situation changes for the replication letters-in-digits experiment.

Section C - Binning Method for high vs low visibility trials

In order to determine which binning method was appropriate for separating the data from[6] into high and low visibility trials, we evaluated the grouped validity for each potential binning method. This showed quite clearly (see figure 3) that the split with the strongest validity was an even split with the 3 lowest visibility ratings forming the low bin, and the 3 highest visibility ratings forming the high bin.

Section D - Subjective experience in the Simultaneous Type/Serial Token model

In this section, we detail how the STST model is used to simulate ERPs, the setup of the STST model used to extract a visibility rating, and how the visibility rating was calculated. Our virtual ERPs are calculated from a computational implementation of the STST model, neural-STST[3,7]. As in the STST model described in the STST model section, the neural STST model is organised as layers of nodes, connected via weighted connections. These connections are the analogue of synaptic projections in the brain, and in order to calculate the P3, we therefore introduce the concept of excitatory post synaptic potential to these virtual nodes. This is calculated as the activation value of the node multiplied by the weight value of its connections to the subsequent layer. The virtual P3 is then calculated as the sum of these excitatory post synaptic potentials across a subset of the layers. We follow previous work in using the 3rd, 4th, 6th and 8th layers of the neural-STST model, corresponding to the item layer, the task filtered layer, the binder gates and the token gates. As in previous work[7], we also implement a retinal delay of a model equivalent of 70ms. Compared to previous works using virtual ERPs from the STST model, we selected a slightly different stimulus range over which to calculate this virtual P3. Specifically, we sample a range of stimulus strengths with greater variability (-0.078 to +0.078 -> -0.1625 to +0.1625), at a slightly higher average stimulus and distractor strength (0.520 -> 0.570). This approach is consistent with previous simulations with the STST model, where we allow input strength ranges to vary reflecting the fact that different experiments being modelled might have quite different stimulus types and sensitivities. Compared to previous iterations of virtual P3 generation, we do not directly sum the components of each item in the stream to create the P3. We instead only consider the contribution to the P3 of a target to the extent that it does not conflict with the P3 of another, active target.

In order to calculate subjective report from these virtual P3's we, as described in the main body of the paper, calculate the number of time steps that a stimulus spends above a given threshold. For the results given in this paper, this threshold is 0.05. Additionally, although this method gives us a continuous subjective report, for the purposes of comparison with the human data from[6], it is necessary to be able to divide these subjective reports into the discrete cases of high/low visibility. Since we are unable to be sure that each lag contains the full range of possible subjective reports, we do this by lag. Since we also do not know how the visibilities are distributed across each lag, but wish to make a simple, even split as far as possible, we use the average as the splitting point for high/low visibility. It is also necessary to normalise these time steps counts into visibility ratings that can be compared to the human data. In the spirit of the simplicity that has driven the creation of the model so far, we simply normalise the timesteps by a linear factor. To keep the range plausible and remain data driven, the value we selected was the most visible stimulus in the entire experiment, and divided each visibility rating by this in order to give a "percentage visibility". In this way, we provide a very simple index of both continuous and binned subjective report that requires no changes to the original model.

Section E - Further SESE model ERPs

In figure 4 we provide some further results comparing human and SESE generated ERPs. This compares Human lag 3 with SESE lag 3, and human lag 3 with SESE lag 4.

Section F - Justifying Non-Monotonic Pattern in Figure 6

It is interesting to note that in the replication (pure letters-in-digits) data set, non-monotonicity goes up when lag-1 is removed:

compare figures 8B and 9B. There are a number of points that can be made about this.

1. The identification of a non-monotonic pattern when lag-1 is excluded is not inconsistent with the attentional and experiential blink curves we observe for this data set – see figure 6B, where the distance between T2 report accuracy and T2 subjective visibility are further apart at lag-2 than at higher lags.
2. Non-monotonicity with lag-1 excluded is not so obvious from figure 6D, although, there is a definite kink for lag-2 relative to lags 3 and 5. Furthermore, small fluctuations in the lag-2 data point, which there certainly are across participants, could create a non-monotonic pattern driven by lag-2.
3. The importance of Lag 1 in the averaged data is not necessarily accurately reflecting each individual. Accordingly, removing the lag-1 point does not consistently effect each individual participant. Although the overall trend is for more evidence for non-monotonicity, 4 of the 12 participants, for example, gain evidence for monotonicity with the removal of the lag 1 data-point, compare figure 8B and figure 9B.
4. Finally, and perhaps most importantly, it is well attested that averaged state-trace curves can fail to be representative of the across participant pattern. Indeed, it could be that the lag-2 point is only at the position shown in figure 6D for the average and not for any of the participants.

References

1. Davis-Stober, C. P., Morey, R. D., Gretton, M. & Heathcote, A. Bayes factors for state-trace analysis. *Journal of mathematical psychology* **72**, 116–129 (2016).

2. Chun, M. M. & Potter, M. C. A two-stage model for multiple target detection in rapid serial visual presentation. *Journal of Experimental psychology: Human perception and performance* **21**, 109 (1995).

3. Bowman, H. & Wyble, B. The simultaneous type, serial token model of temporal attention and working memory. *Psychological review* **114**, 38–70 (2007). LR: 20070117; CI: ((c) 2007; JID: 0376476; ppublish.

4. Wyble, B., Bowman, H. & Nieuwenstein, M. The attentional blink provides episodic distinctiveness: sparing at a cost. *Journal of Experimental Psychology: Human Perception and Performance* **35**, 787 (2009).

5. Morris, C. N. Parametric empirical bayes inference: theory and applications. *Journal of the American Statistical Association* **78**, 47–55 (1983).

6. Pincham, H. L., Bowman, H. & Szucs, D. The experiential blink: Mapping the cost of working memory encoding onto conscious perception in the attentional blink. *Cortex* **81**, 35–49 (2016).

7. Craston, P., Wyble, B., Chennu, S. & Bowman, H. The attentional blink reveals serial working memory encoding: Evidence from virtual and human event-related potentials. *Journal of cognitive neuroscience* **21**, 550–566 (2009).

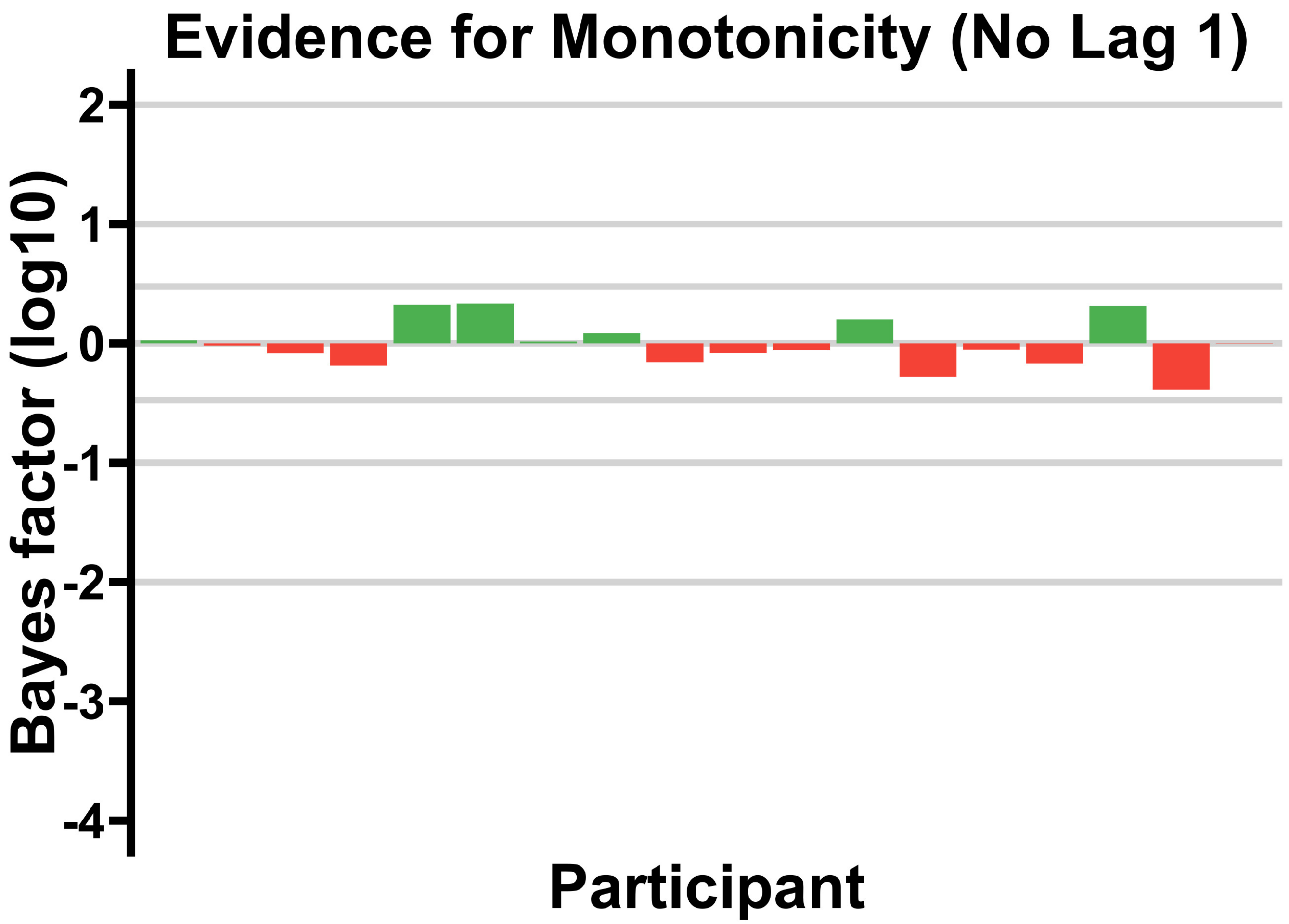

Figure 1. Respective monotonicity vs non-monotonicity for the original (colour-marked T1) dataset excluding the lag 1 datapoint. Results are weak and strongly hetrogenous, with grouped (not log) $BF_{(M/NM)|D} = 6.69 \times 10^{-1}$. This essentially provides no evidence either way for monotonicity, a strong constrast to the analysis with the Lag 1 datapoint included, which finds a strongly non-monotonic effect with grouped (not log) $BF_{(M/NM)|D} = 1.17 \times 10^{-17}$

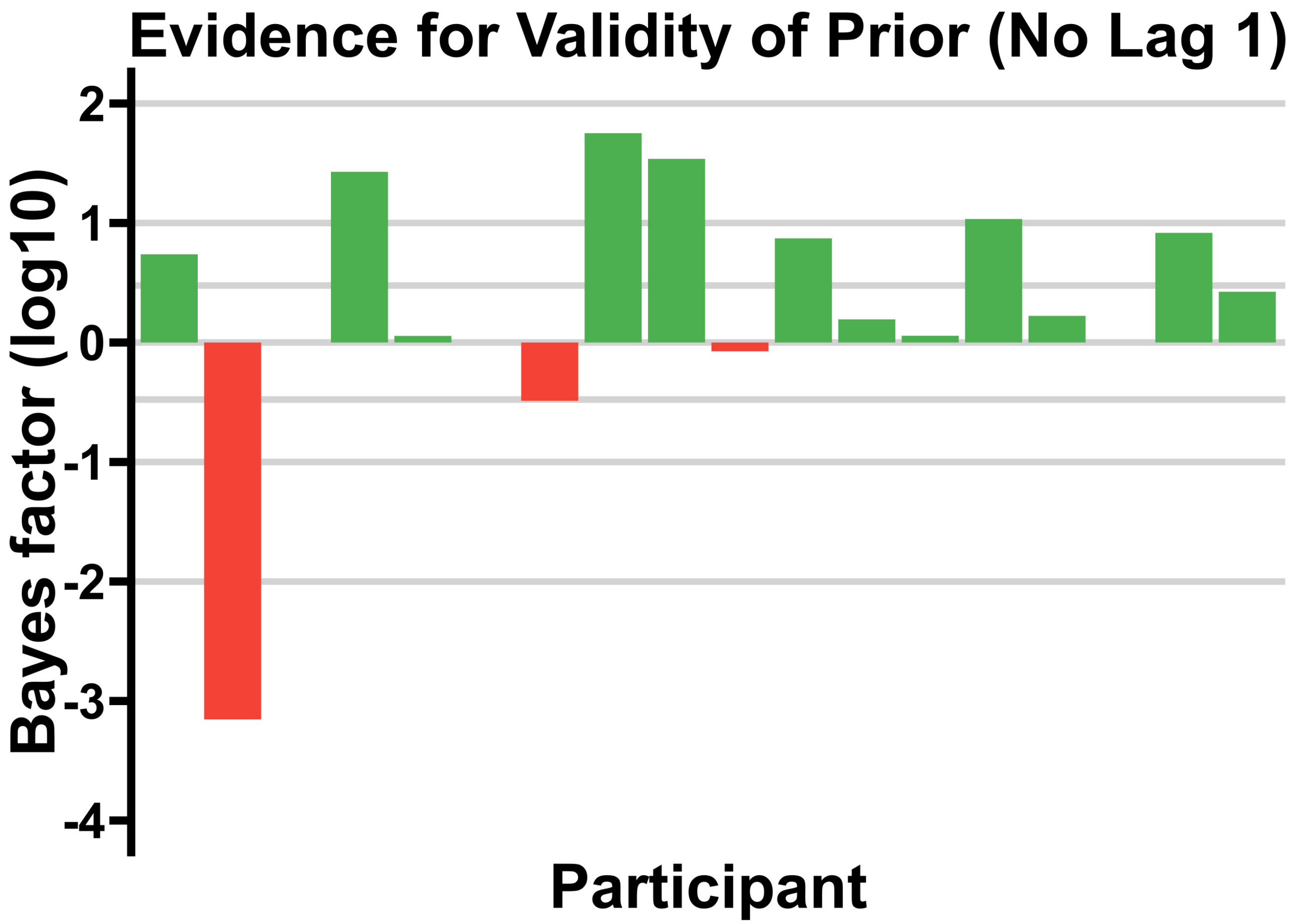

Figure 2. Validity for each participant for the set of prior constraints derived from the original using our empirical prior method, for the original (colour-marked T1) task dataset excluding the Lag 1 datapoint. Any constraints no longer valid without Lag 1 have been removed. At the group level, the evidence is strongly in favour of the constraints fitting the data, with grouped (not log) $BF_{D/N(D)} = 1.01 \times 10^{13}$, extremely close to the grouped validity with Lag 1 included with grouped (not log) ($BF_{D/N(D)} = 1.07 \times 10^{13}$.

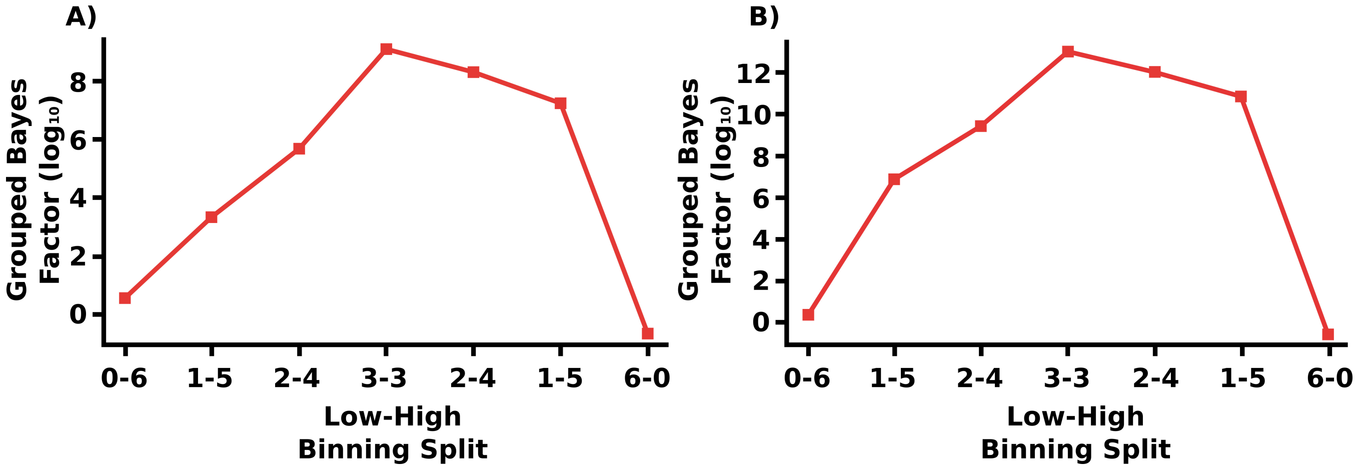

Figure 3. A) Grouped Bayes factor for validity of the original (colour-marked T1) dataset across each potential binning method for high and low visibility using the original set of constraints based on the data from (Nieuwenhuis, de Kleijn 2011). B) Grouped Bayes factor for validity across each potential binning method for the empirical prior constraints.

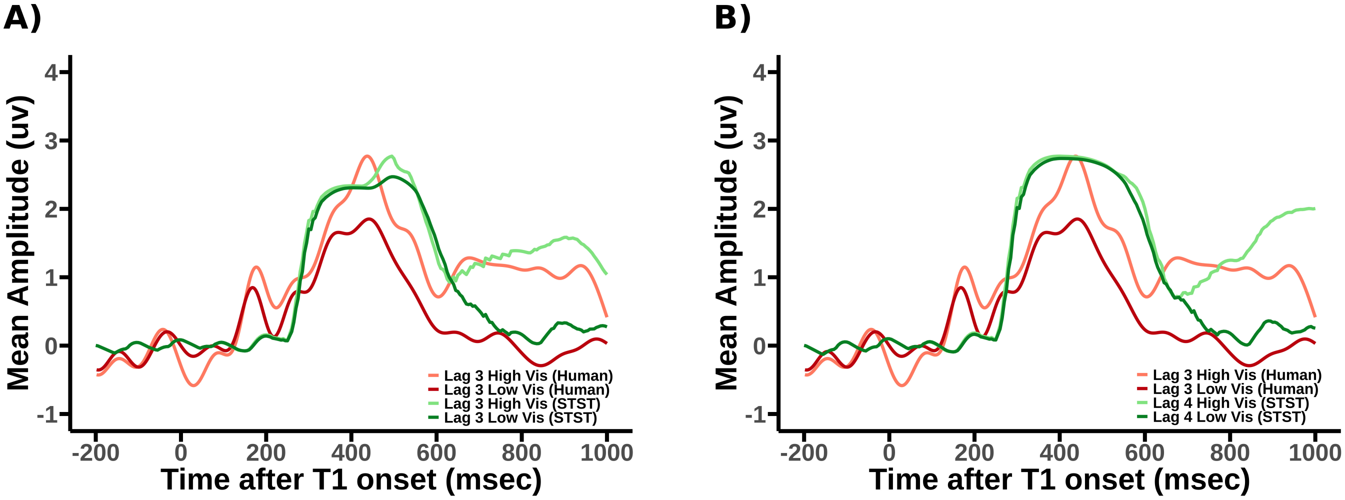

Figure 4. A comparison, for both high and low T2 visibility, given correctly reported T1, of the human ERPs from the original colour-marked T1 data analysis[6]. A) Lag 3 Human ERPs vs Lag 3 STST virtual ERPs. B) Lag 3 Human ERPs vs Lag 4 STST virtual ERPs. Importantly, as previously discussed, neither the function or the structure of the STST model, as given in[3] were changed when generating the virtual P3s. Note that the human ERPs presented are slightly different to those from[6], as ours exclude order errors to be consistent with previous sections.
